# Mechanical Properties of 3D Nanostructures Obtained by Focused Electron/Ion Beam-Induced Deposition: A Review

**DOI:** 10.3390/mi11040397

**Published:** 2020-04-10

**Authors:** Ivo Utke, Johann Michler, Robert Winkler, Harald Plank

**Affiliations:** 1 Laboratory for Mechanics of Materials and Nanostructures, Empa-Swiss Federal Laboratories for Materials Science and Technology, CH-3602 Thun, Switzerland; johann.michler@empa.ch; 2 Christian Doppler Laboratory for Direct-Write Fabrication of 3D Nano-Probes (DEFINE), Institute of Electron Microscopy and Nanoanalysis, Graz University of Technology, 8010 Graz, Austria; robert.winkler@felmi-zfe.at (R.W.); harald.plank@felmi-zfe.at (H.P.); 3 Institute of Electron Microscopy and Nanoanalysis, Graz University of Technology, 8010 Graz, Austria; 4 Graz Centre for Electron Microscopy, 8010 Graz, Austria

**Keywords:** nanoscale additive manufacturing, gas-assisted electron and ion-induced deposition, focused electron beam-induced deposition (FEBID), focused ion beam-induced deposition (FIBID), mechanical properties, Young’s modulus, hardness, yield strength, fracture strength, quality factor, density, nanogranular material, metal matrix material, carbon, polymer, glassy carbon, graphitic carbon, amorphous hydrogenated carbon, diamond-like carbon

## Abstract

This article reviews the state-of-the -art of mechanical material properties and measurement methods of nanostructures obtained by two nanoscale additive manufacturing methods: gas-assisted focused electron and focused ion beam-induced deposition using volatile organic and organometallic precursors. Gas-assisted focused electron and ion beam-induced deposition-based additive manufacturing technologies enable the direct-write fabrication of complex 3D nanostructures with feature dimensions below 50 nm, pore-free and nanometer-smooth high-fidelity surfaces, and an increasing flexibility in choice of materials via novel precursors. We discuss the principles, possibilities, and literature proven examples related to the mechanical properties of such 3D nanoobjects. Most materials fabricated via these approaches reveal a metal matrix composition with metallic nanograins embedded in a carbonaceous matrix. By that, specific material functionalities, such as magnetic, electrical, or optical can be largely independently tuned with respect to mechanical properties governed mostly by the matrix. The carbonaceous matrix can be precisely tuned via electron and/or ion beam irradiation with respect to the carbon network, carbon hybridization, and volatile element content and thus take mechanical properties ranging from polymeric-like over amorphous-like toward diamond-like behavior. Such metal matrix nanostructures open up entirely new applications, which exploit their full potential in combination with the unique 3D additive manufacturing capabilities at the nanoscale.

## 1. Introduction

**Figure 1:**
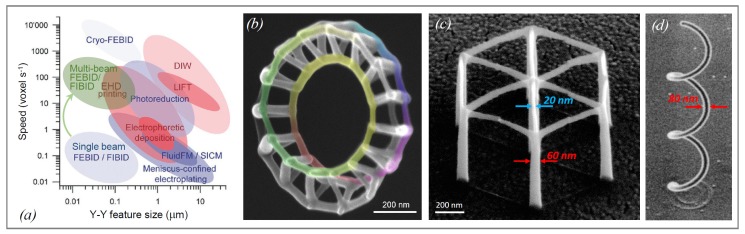
(**a**) Comparison of 3D nanoprinting via focused electron beam-induced deposition (FEBID) and focused ion beam-induced deposition (FIBID) to other sub-10-μm metal additive manufacturing methods within the speed-size parameter space. The acronyms stand for direct ink writing (DIW), electrohydrodynamic printing (EHD), laser-induced forward transfer (LIFT), the electroplating of locally dispensed ions in liquid–atomic force microscopy (AFM), and cantilever-based (FluidFM) and glass capillary-based scanning ion conductance microscope (SICM). The potential of multi-beam versus single beam FEBID/FIBID is indicated. Modified from Hirt et al. [1]. (**b**,**c**) Examples of 3D FEBID. (**b**) Reprinted from Winkler et al. [7], with the permission of AIP Publishing. (**d**) Example of 3D FIBID structure. Modified from Matsui et al. [8].

In a recent review of the additive manufacturing of metal structures, Hirt et al. [1] comprehensively reviewed the state-of-the-art concerning additive 3D manufacturing methods of metal structures at the micron scale, mesoscale and nanoscale. As a main result, focused electron beam and focused ion beam-induced deposition (FEBID and FIBID) were found to enable the smallest lateral feature sizes, see Figure 1a, as well as enormous flexibility in 3D designs. FEBID and FIBID are sequential nanoprinting techniques and thus suffer from low throughput when performed on single electron and ion beam machines. However, multi-beam FEBID with 196 beams in one microscope was already demonstrated by Post et al. [2] as well as FEB and FIB microscopes with >10^5^ individually addressed beams [3,4], which would allow boosting the overall speed accordingly as indicated (for 1000 beams) in Figure 1a. Another well-known 3D printing technology is two-photon lithography [5,6] in liquid polymer photoresists. Mechanics studies of the sub-micron-scale, lightweight, high-strength polymer 3D architectures briefly discussed in Section 4 are mainly based on this technology. Although it offers good resolution in the 200 nm range and high throughput, it seems that direct extension to mask-free printing of metal architectures is prohibitive due to strong laser light absorption. 

The main aim of this article is to give an overview of the experimentally confirmed mechanical properties of FEBID and FIBID materials and to relate them to their structural and compositional characteristics, which in turn will depend on their deposition (irradiation) parameters. We critically review the methods employed including fundamental FEBID and FIBID phenomena, such as the trajectories of electrons and ions in high-aspect ratio pillar deposits and related core–shell structures, as well as the mechanical measurements with their governing equations and precision. Mechanical properties will include the elastic moduli, hardness, quality factors (for resonating structures), plastic yield, fracture strength/strain, and density. Most of the properties were measured for as-grown FEBID and FIBID material, but we also include recent exciting developments of three-dimensional structures with sub-100-nm unit features and post-growth processing such as electron beam curing and atomic layer deposition coatings, which show big potential in tuning mechanical (and other functional) properties of FEBID and FIBID 3D nanoprinted structures.

## 2. Methods and Materials

### 2.1. 3D Nanoprinting via FEBID & FIBID

**Figure 2:**
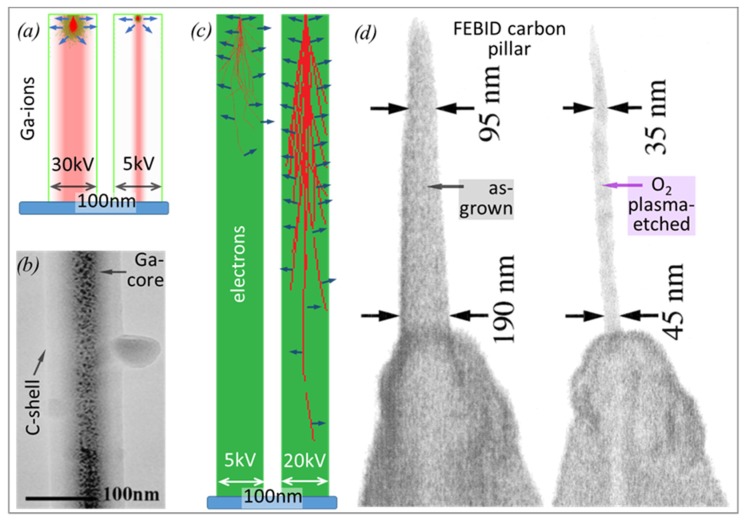
(**a**) SRIM (stopping and ranges of ion in matter) [15] simulation of a zero-diameter gallium ion beam and 100 trajectories (red) with the adjacent collision cascade (green) in a 100 nm wide and 400 nm tall pillar of carbon (density 2 g/cm^3^) illustrating the inevitable implantation of Ga (shaded red) and its redistribution via the collision cascade for primary energies of 30 kV (left) and 5 kV (right). Blue arrows signify secondary electrons (SEs), leading to deposition events if generated in close proximity to the surface. (**b**) TEM image of a core–shell structure of an as-grown Ga-based, FIBID carbon pillar using phenanthrene. Modified from Matsui et al. [69]. (**c**) CASINO [66] simulation of a zero-diameter electron beam and 20 trajectories (red) in a 100 nm wide and 1 μm tall carbon pillar for primary electron energies of 5 keV (left) and 20 keV (right). The electron trajectories were truncated once they exit the pillar volume for the sake of clarity. Blue arrows again indicate SEs generated along the electron paths. (**d**) SEM side view images of a carbonaceous FEBID pillar deposited from residual vacuum pump oil molecules on an AFM tip before (left) and after oxygen plasma treatment (right). The much narrower core that remains indicates a different chemical bonding, which is closely related to different mechanical properties. Modified from Wendel et al. [68]. Note that (**a**) and (**c**) have the same scale for easy comparison and that the pillar diameters will also depend on the charged particle beam intensity profile (focus), which was omitted for the sake of clarity.

At the heart of FEBID and FIBID are electron/ion-triggered dissociation reactions of surface adsorbates, which form the local deposit [9,10]. The volatile, physisorbed surface adsorbates are continuously replenished within the irradiated area by gas supply from a gas injection system (GIS) as well as by surface diffusion from the area around the irradiated spot. The surface dissociation reaction is widely non-thermal and thus localized to the irradiated area of the finely focused beam, including subsequently generated electron species, namely backscattered electrons (BSE), forward scattered electrons (FSE) for taller structures, and, in particular, related secondary electrons (SE) in close proximity, which have the highest cross-section [11]. Molecule dissociation by electrons follows different channels: dissociative electron attachment, dissociation into neutrals, and—the most popular—is fragmentation into ions, which is conceptually used and widespread in mass spectrometry to determine the composition of gases and materials [12]. Whatever dissociation mechanisms are finally active, volatile dissociation fragments will leave the surface again and are pumped away, while the non-volatile fragments will form the intended deposit. The non-dissociated precursor desorbs after a short residence time, which therefore limits the deposition of material only to the beam impact region, in contrast to other methods, such as chemical vapor deposition [13] or atomic layer deposition [14] using similar precursor materials. By that, the electron/ion beam acts as a functional nanopen. Focused ion beams (FIBs) also generate SEs, which can dissociate surface-adsorbed molecules, but they also generate confined collision cascades within the surface volume below the irradiated area. Parts of the collision cascade atoms are sputtered away when having sufficient kinetic energies to overcome the surface binding of a few electron volts [10,15]. However, a greater amount of collision cascade atoms have lower energy and end up as excited surface atoms, which can transfer their energy to surface adsorbates and initiate their dissociation. For gallium ions and most of the other focused ion beam species, such as He, Ne, and Xe used nowadays, the dominant adsorbate dissociation mechanism is via excited surface atoms within the collision cascade area [16] of the impinging primary ion beam, while SE-triggered dissociation defines the material and shape of 3D deposits at its periphery [17]; see also Figure 2. For a more detailed discussion of the dissociation mechanisms in FEBID and FIBID, the reader is referred to the referenced literature [11]. A final comment regarding the non-thermal dissociation of adsorbate molecules concerns the scission bond selectivity. Generally, with the energy spectrum of SEs and excited surface atoms in the electron volt (eV) range, together with the primary electron (PE)/BSE energies close to the initial energies, typically in the kilo electron volt (keV) range, there can be no preferential bond scission in the molecule expected. This coincides with the experimental finding that gas-assisted ion and electron beam deposit materials often contain all the constitutional atoms of the precursor molecule [18,19]; see also Table 1 for the deposit composition of mechanically investigated materials. However, there are exceptions, and high purity material of cobalt, iron, gold, and silver can be deposited directly [20,21,22,23,24,25]. Furthermore, for these and other metals, current research activities successfully concentrate on novel precursor design and in situ or post-growth purification techniques [26,27,28,29,30,31,32,33,34] and their simulation [35].

The volatile precursors as well as the composition range of FEBID and FIBID materials for which mechanical properties were reported are summarized in Table 1. A general observation from Table 1 is that the mechanical properties were not yet measured for all of the highest metal content FEBID and FIBID materials. This is due to the relatively recent and still ongoing exciting developments in pure metal FEBID and FIBID, the latter especially with noble gas ions, which do not entail an additional but unwanted implantation by the ion species. A straightforward consequence is that the mechanical properties for low metal content material will be dominated by the matrix properties. This will be discussed in Section 3.1.2.

Another observation from Table 1 is that the hydrogen content in the FEBID and FIBID obtained material is generally not specified, although this value is of great importance to relate the mechanical properties of these carbon deposits, which can vary from very compliant and soft (polymers) to extreme hard and stiff (diamond) due to their composition. The reason for this lack of characterization is that hydrogen detection, e.g., via elastic recoil detection analysis (ERDA), preferably requires centimeter-sized thin film areas, which is currently unattainable for nanoprinting by single focused beam-based FEBID and FIBID. 

Turning to physical deposit parameters, the 3D deposit is shaped by the computer-controlled movement of the focused electron/ion beam, by the spatial beam profile, i.e., focus conditions, as well by the interaction volume of the charged particles within the printed nanostructure. For a detailed review, the reader is referred to a recent article by Winkler et al. [65]. The interaction volumes of gallium ions and electrons in carbon pillar material were simulated by SRIM (stopping and ranges of ion in matter) [15] and CASINO (Monte Carlo simulation of electron trajectories) [66] and are shown in Figure 2 together with real pillar deposition experiments. For the simulations shown in Figure 2, we have chosen typical conditions for most FEBID and FIBID experiments (incident-charged particle energies between 5 and 30 keV and a carbon pillar diameter of 100 nm). 

The striking difference between FEBID and FIBID concerns the interaction volume of the incident charged particles, which is one to two orders of magnitudes smaller for the latter, as ions lose their energy much faster than electrons (see [10]). In carbon, the lateral expansions of FIB interaction volumes are below 20 nm, even at high primary energies of 30 kV, as indicated in Figure 2a. Furthermore, gallium ions are incorporated within the growing pillar and lead to a gallium-rich core, as shown by a transmission electron microscopy (TEM) micrograph in Figure 2b. The diameter of that gallium-rich core scales with the primary ion energy, as shown by the scheme in Figure 2a. Furthermore, the collision cascade of the substrate atoms is much larger at higher incident ion energy and will further contribute to the spreading of the implanted gallium. The dissipated energy-per-ion is equal to its incident energy (given by the acceleration voltage), as the energy carried away by backscattered primary ions is zero (there are no backscattered gallium ions) and the energy removed by sputtered atoms is negligible. [15] 

For electrons, in contrast, the interaction volume in carbon is much larger than the here assumed 100 nm wide pillars; it is roughly about 1 μm and 8 m at 5 keV and 20 keV, respectively. All the impinging electrons will enter the pillar apex within the focus spot area of the electron beam and then spread and dilute until they exit the pillar in the backward (BSE) or forward (FSE) direction, as schematically shown in Figure 2c. The broadening of the primary electron beam within the tip apex is responsible for the conical pillar apex shape, as shown in Figure 2d and shown in slightly more detail in another review article by Plank et al. [67]. The pillar also grows laterally due to SE generation close to the surface, which emerges due to the inelastic collision events along the primary electron trajectories. Interestingly, also for FEBID, a core–shell structure becomes visible when the as-grown carbon pillar is subjected to oxygen plasma, although it is not due to extra implanted material as for focused ion beams. In Figure 2d, a slim core of more plasma-resistant and hard carbon material withstands the plasma attack (a distinctly slow etch rate), while the softer carbon shell material was removed [68]. This observation can be explained when having a look at the density of inelastic scattering events versus the penetration depth in Z, which decreases for deeper regions. Consequently, there is a radial distribution of the sp^2^/sp^3^ carbon hybridization ratio as well as that of hydrogen, which impact the mechanical properties, as discussed in Section 3.1.1. The lateral distribution of those variations inherently depends on the primary electron energy, as schematically evident in Figure 2c. For an excitation volume being larger than the pillar volume, the overall implanted energy W can be estimated as [10]

(1) $$W\approx \left(\frac{dE}{ds}\right)\Delta s$$

where the term dE/ds denotes the energy loss along the particle trajectory (the so-called stopping power) and Δs denotes the average trajectory length of the incident particle. 

As can be seen in Figure 2c, the average electron trajectory length inside the pillar increases with higher energy; however, the electron loss per trajectory length for electrons is inversely proportional to its actual energy, as derived in the well-known Bethe formula [70]; for 5 and 20 keV incident energy, the stopping power in carbon is about 0.3 eV/Å and 0.1 eV/Å, respectively. With average electron trajectory lengths of about 170 nm (5 keV) and 400 nm (20 keV), the implanted energy is thus roughly 500 eV and 400 eV per 5 keV and 20 keV incident electrons, respectively. Thus, the implanted energies for 5 and 20 keV incident electrons into the same slim pillar are the reverse to what the incident electron energy ratio would let us expect. However, when the deposit volume is a large carbon cube with dimensions comparable to the electron ranges, the implanted energy would then simply scale with the ratio of incident electron energies, as it was the case for the ions. However, for the small model structures and incident electron and ion energy ranges considered in Figure 2, electrons dissipate 10 to 100 times less energy than gallium ions into 100 nm diameter carbon pillars for the same acceleration voltage and beam current. Consequently, this leads to differing irradiation effects and consequently, the resulting collision cascade for ion irradiation will entail a strong amorphization of any deposited material.

Another implication of energy implantation into a growing pillar structure is the generation of heat at the pillar apex. The temperature difference ΔT between the pillar apex and base (at room temperature) can be estimated [10,71]

(2) $$\Delta T=P\cdot \frac{l}{\pi r^{2}}\cdot \frac{1}{\kappa }$$

with l, r, and κ being the pillar length, its average radius, and the heat conductivity of the pillar, respectively. For deposit volumes larger than the excitation volume of the charged particles, the implanted power $P=U_{acc}\left[V\right]\cdot I_{p}\left[A\right]$, with $I_{p}$ representing the beam current and $U_{acc}$ representing the acceleration voltage. If the pillar diameter is smaller than the excitation volume, then $P=W\left(I_{p}/e_{0}\right)$, with W defined by Equation (1) and $e_{0}$ being the elementary charge. For a beam current of $I_{p}=$1 pA and an acceleration voltage of 5 kV and 30 kV, the implanted power is 5 nW and 30 nW, respectively, when working with ions. For FEBID, the same beam current leads to an implanted power of 0.5 nW (5 kV) and 0.4 nW (30 kV), which are orders of magnitude lower. Now assuming a 100 nm wide and 10 m long carbon pillar with an average thermal conductivity of $\kappa =1\;Wm^{-1}K^{-1}$, Equation (2) suggest a temperature increase of 6.4 K (5 kV) and 38 K (30 kV) when working with Ga ions. For electrons, in contrast, the temperature rise lies in the range of 0.6 K for low and high primary electron energies. For comparison, the typical thermal conductivities of hydrogenated amorphous carbon are in the range of $\kappa =0.2\;Wm^{-1}K^{-1}$, and for diamond-like amorphous carbon, it is $\kappa =2.2\;Wm^{-1}K^{-1}$ [72] (for comparison, crystalline diamond has $\kappa =1000-2200\;Wm^{-1}K^{-1}$). In other words, depending on the carbon material deposited, the temperature rise on the pillar top can be up to five times higher and reach 192 K, meaning the top has a temperature of 217 °C compared to the pillar base at a room temperature of 25 °C. Increasing the beam current will also linearly increase the pillar apex temperature. Such high temperatures can affect the dissociation and in particular the desorption frequency and diffusivity of adsorbed molecules as well the mobility of already deposited molecule fragments and elements, which allows them to rearrange into metal grains or carbon networks, or to diffuse out in the case of volatile products such as hydrogen from the hydrocarbon molecules. Please note that the aforementioned considerations apply for vertical pillars. When fabricating more complex 3D architectures with overhanging features [65,73,74,75], localized beam heating has been identified as the main effect concerning the fabrication precision, as recently revealed by Fowlkes et al. [76,77], which also has implications on the chemical state of the matrix and by that on the mechanical properties. However, as heating depends on the design and the feature diameters, one would prefer to have no temperature differences during growth in order to spatially maintain the mechanical properties. Since metals have very good heat conductivity in the range of $\kappa \approx 500\;Wm^{-1}K^{-1}$, being more than two orders of magnitude better than the above-discussed carbon material, the FEBID and FIBID of metallic nanostructures run smoothly without large temperature changes at the growth front, as already observed for the nominally pure Co_3_Fe deposits. [78]

Aside from sole temperature effects, the working regime during FIBID and FEBID can also lead to variations in the morphologies, chemical composition, and functional properties including mechanical parameters. In principle, there are two extremes, namely an electron/ion limited (particle limited regime, or PLR) and molecule limited regime (MLR). Following the notation, PLR conditions mean an excess of precursor molecules, which is favorable for the highest precision and smallest feature sizes. In contrast, MLR conditions provide more particles than molecules, where the former can lead to additional processes such as matrix modifications, although feature sizes are basically larger [10,79,80]. The exception for the latter are taller, freestanding 3D objects, where strongly tilted elements can reveal widths down to the sub-20-nm regime [73,74]. The underlying processes of such situations are related to the convolution of the relative angle between the growing element and the incoming electron beam, the applied primary energies, and the shift to gas flux adsorption-dominated replenishment, leading to varying cross-sectional profiles [65]. As the latter is actually unwanted due to the strong design dependency, current activities focus on the compensation by advanced 3D patterning strategies, as discussed in another article in this special issue [76,77].

### 2.2. Mechanical Characterization

Here, we will shortly review methods of measuring the elastic modulus (or Young’s modulus), hardness, yield strength for plastic deformation and fracture, and density of FEBID and FEBID materials. Before starting, we must note that the Mechanics community and the FEBID/FIBID community developed their own terminology: the FEBID/FIBID community refers to "pillars" as high aspect ratio cylindrical structures grown on a substrate typically in the spot exposure mode. However, in the Mechanics community, one-end-clamped high aspect ratio structures are denoted as "cantilevers" or "beams", which are frequently used in bending, buckling, and vibration experiments, while a pillar has a low aspect ratio and is used for (nano/micro) compression experiments. 

In this article, we will adopt the term pillars for FEBID/FIBID material regardless of high or low aspect ratio and understand that readers will capture the context from the measurement method. 

Most of the measurements will require scanning electron microscopy (SEM) integrated mechanical setups to identify and situate the pillars, to apply the force (load) correctly, and to observe the specimen’s response. Commercial systems are compatible with most SEMs, FIB microscopes or SEM/FIB dual beam microscopes comprise nanoindentors having force (load) and displacement readouts; these are operated in a force or displacement-driven fashion. Furthermore, SEM-integrated AFM systems with cantilever-based force sensors detect the deflection of the AFM cantilever via a piezoresistive signal, which is calibrated via the cantilever’s force constant to access the arising force in a quantitative manner. The SEM operator directly observes the displacement of the pillar during or after the experiment. Alternatively, precise stage control can be used to position a calibrated cantilever ($k_{c}$) against the structure of interest while measuring the cross-section $A_{D}$ and the compression Δl of the pillar together with the AFM cantilever displacement $\delta_{c}\;$to calculate the exerted force F via $k_{c}\cdot \delta_{c}$ and the elastic modulus, as described in the following section. 

#### 2.2.1. Elastic Modulus

**Figure 3:**
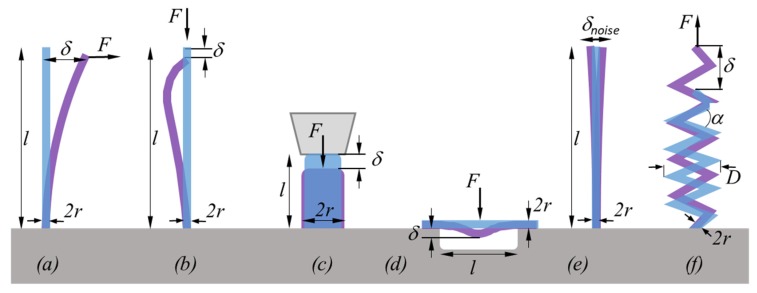
Approaches to measure the elastic modulus of FEBID/FIBID materials using simple high/low aspect ratio pillar or spring geometries. (**a**) Bending (one end pinned), (**b**) buckling, (**c**) nanocompression, (**d**) bending (two ends pinned), (**e**) thermal noise vibrations, and (**f**) spring tension (or compression). Circular cross-sections enable simple mathematics (see text), yet complex 3D architectures can be measured according to (**a**)–(**f**) as well.

The elastic modulus *E* can be determined in dependency on the observed geometry—namely bending, buckling, tensile, or compression experiments on pillars or springs, as schematically shown in Figure 3. An FEBID/FIBID nanostructure has a certain stiffness (spring constant) k, which resists against the deformation (or displacement) δ brought about by the force F acting on it. Within the Euler–Bernoulli theory, the stiffness of uniform cross-section pillars is usually a function of the elastic modulus and the geometry of the nanostructure and is generally given as $k=3EI/l^{3}$ [81,82]. With the known force equilibrium F=k·δ, one can derive the general expression for the elastic modulus

(3) $$E=\frac{l^{3}}{3I}\frac{F}{\delta }\;,$$

where l is the length of the uniform pillar and I is the second moment of area about the neutral axis (or area moment of inertia), which can be found in textbooks for various pillar cross-sections.

The force F can be applied in different ways: (i) by SEM-integrated indenters/cantilevers with force-readout and displacement control (δ), which allows also for convenient in situ force versus displacement monitoring, (ii) by flexible cantilevers that allow calculating the exerted force by their own deflection and cantilever force constant, or (iii) by electrostatic fields, which needs a pre-calibration step [47]. For uniform beams with an ideal circular cross-section of constant radius (*r*) and density (ρ) over their length (*l*), the area moment of inertia $I=\pi r^{4}/4$ and the following relations hold:

(4) $$E=\frac{4l^{3}}{3\pi r^{4}}\frac{F}{\delta }\;\mathrm{for}\;\mathrm{one}\;\mathrm{end}\;\mathrm{pinned}\;\mathrm{bending}$$

(5) $$E=\frac{4{\left(K\cdot l\right)}^{2}\cdot F}{\pi^{3}r^{4}}\;\mathrm{for}\;\mathrm{buckling}$$

(6) $$E=\frac{l}{\pi r^{2}}\frac{F}{\delta }\;\mathrm{for}\;\mathrm{uniaxial}\;\mathrm{nanocompression}\;\mathrm{without}\;\mathrm{buckling}$$

(7) $$E=\frac{l^{3}}{\pi r^{4}}\frac{F}{\delta }\;\mathrm{for}\;2\;\mathrm{ends}\;\mathrm{pinned}\;3-\mathrm{point}\;\mathrm{bending}.$$

The constant K in Equation (5) depends on the buckle geometry and fixation and can vary from 0.5 to 1 [83,84]. For beam geometries with different cross-sectional geometries, such as tube, rectangle, elliptic, or core–shell, Equations (4)–(9) would slightly change to adapt for the varying geometrical moment of inertia. As the pillar dimensions enter with power dependencies into the calculation of the elastic modulus, they need to be characterized as precise as possible to obtain reliable values from these experiments, since their relative errors sum up with the degree of the power exponent. For example, the error propagation for E in Equation (4) yields |ΔE/E|=3·|Δl/l|+4·|Δr/r|+|ΔF/F|+|Δδ/δ|, meaning that 5 rel.% relative error for nanopillar dimensions end up in 35 rel.% for E. If the precision of the force and displacement readouts are assumed to be the same in all approaches of Figure 3, then the nanocompression test is the least exposed to readout errors of pillar dimensions by SEM. However, to avoid artifacts by unwanted buckling, the aspect ratio for nanocompression experiments should be not much larger than 1:2 (diameter to length). Of general note is that the relative errors Δr/r and Δl/l become more pronounced when the absolute radii r and absolute lengths l get into the lower nanoscale. Furthermore, exerting the force perfectly perpendicular to surfaces or parallel to the pillar axis can be very challenging in real experiments, which requires further trigonometric corrections during the calculation of E, as comprehensively discussed in a book chapter of Friedli et al. [85]. For situations where e.g., pillar geometries reveal irregular deviations from perfect cylindrical shapes, finite element simulations are needed to obtain correct results. 

**Figure 4:**
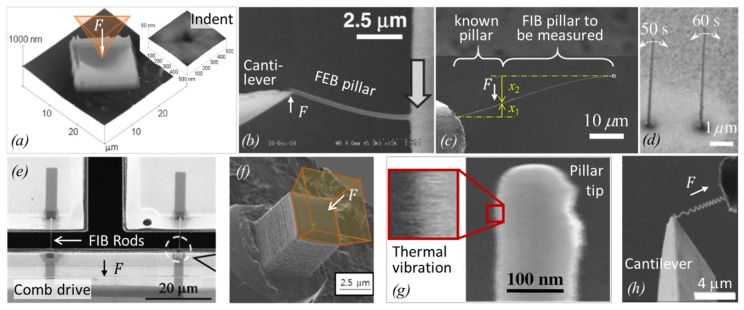
Selected examples of published deposit shapes and methods to determine the elastic modulus. The force *F* acting on the structure is indicated in all images. (**a**) Bulk FEBID square deposit for nanoindentation, modified from Ding et al. [45]. (**b**) FEBID pillar bending by commercial silicon cantilever, modified from Okada et al. [36]. (**c**) FIB pillar bending by another FIB pillar with known spring constant, modified from Guo et al. [37]. (**d**) FIB pillar vibration method combined with density determination, modified from Kometani et al. [38]. (**e**) Tensile strain experiment with horizontal FIB rods on a comb drive stage, modified from Kiuchi et al. [39]. (**f**) Compression of a thick FIB cube with 3.5 μm × 3.5 μm base area, modified from Kim et al. [41]. (**g**) Thermal noise vibration method at the very end of a 21.7 μm long FIB pillar. The inset shows the related fuzzy secondary electron signal, modified from Nonaka et al. [42]. (**h**) Tensile strain experiment with FIB helix fixed between a cantilever and gold glass capillary, modified from Nakamatsu et al. [43]. The organic precursors used were paraffin (**a**) and phenanthrene (**b**) to (**h**).

Figure 4 shows a compilation of experiments with FEBID and FIBID structures following the principles introduced in Figure 3. 

An elegant way to determine the elastic modulus of long pillars without any force actuator is to measure the distribution of pillar deflections caused by thermal noise excitation [42,44,86,87]. Using the spot mode electron beam on the top side edge of the vibrating pillar, the modulated SE signals corresponding to the pillar vibration can be obtained. Using a spectrum analyzer, the amplitude intensity distribution (amplitude versus frequency) is obtained, which peaks at the fundamental pillar frequency and decays with a Gaussian variance $\sigma^{2}$, entering into Equation (8):

(8) $$E=\frac{4l^{3}}{3\pi r^{4}}\frac{k_{B}T}{\sigma^{2}}\;\mathrm{for}\;\mathrm{thermal}\;\mathrm{noise}\;[42].$$

This method requires thermal noise vibrations large enough to be detectable as *blur* in SEM images, as representatively shown in Figure 4g. As an example, pillars with diameters of about 110 nm, elastic moduli around 120 GPa, and vibrating at their fundamental resonance around 2 MHz needed to be taller than 6 μm. Below that, the vibration became undetectable via SEM [87]. A potential source of errors in this approach may be slight off-normal pillar tilts with respect to the substrate, which may affect the amplitude distribution. 

More complicated helix nanostructures were also used to determine the elastic modulus, as shown in Figure 4h. The corresponding Equation (9) is more complicated, although it is still analytical:

(9) $$E=2\left(1+\nu \right)\frac{D^{3}n}{2r^{4}}\left[1-\frac{3r^{2}}{4D^{2}}+\frac{3+\nu }{2\left(1+\nu \right)}\tan^{2}\alpha \right]\frac{F}{\delta }\;\;\mathrm{for}\;1\;\mathrm{end}\;\mathrm{pinned}\;\mathrm{helix}\;$$

with *D*, *r*, and *α* as shown in Figure 3f and *n* representing the number of helix turns. While in this experiment the displacement can be relatively large, which simplifies the quantitative observation, the implication of the Poisson ratio ν adds another source of uncertainty, as this value is so far unknown for FEBID and FIBID material. 

Summarizing, there are six methods for elastic modulus determination employed in the literature so far, which are grouped into (i) nanocompression and nanoindentations requiring large sample volumes (several cube micrometers) and force/distance sensors, and (ii) various bending concepts and the buckling method requiring high aspect ratio pillars. The bending concepts comprise various actuation approaches (mechanical, electrostatic, thermal noise) and freestanding geometrical arrangements (one and two end pinned versus freely rotating, straight pillar, and spirals). Involving vibration experiments as in Figure 4d necessitates the independent determination of the density; see Section 2.2.3. The analysis of the bending and buckling results should include a careful consideration of uncertainties related to the observation of the pillar shape (diameter, length), as these enter with power relations into the elastic moduli equations. Furthermore, deviations from the idealized cylinder structure, such as diameter or mass variations over pillar length, will necessitate finite element simulations. 

#### 2.2.2. Hardness

**Figure 5:**
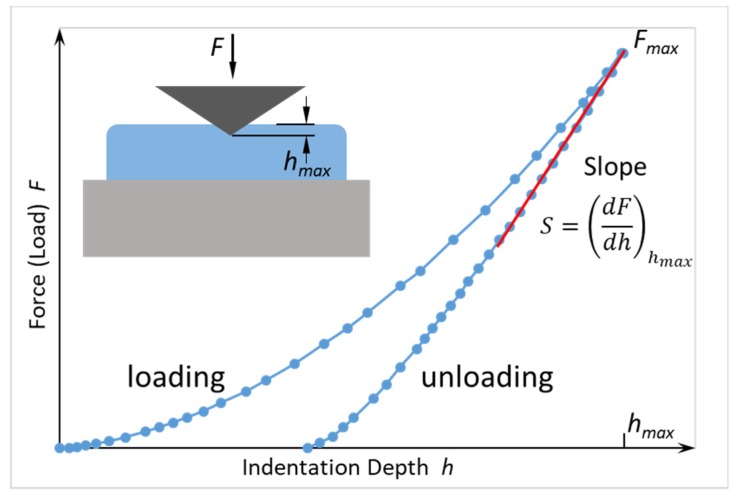
Typical nanoindentation curve for a load–unload cycle. The elastic modulus and hardness derive from the unloading part. The inset schematically shows the indenter tip pushed into the sample.

**Figure 6:**
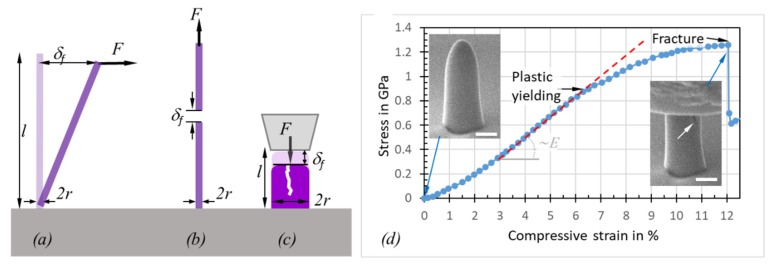
Approaches to measure the yield strength (plasticity) and the fracture strength of FEBID and FIBID materials. (**a**) Bending (one end pinned), (**b**) tensile straining, and (**c**) nanocompression with a flat blunt indenter. An example of a stress–strain diagram obtained from a nanocompression experiment with an FEBID pillar is shown in (**d**). Plasticity and fracture onsets are indicated by arrows; see also the crack propagating into the pillar. Circular cross-sections enable simple mathematics (see text); yet more complex 3D architectures can be also measured according to (**a**)–(**c**).

Hardness and the elastic modulus can be measured by nanoindentation experiments on FEBID and FIBID samples with footprints of several micrometers and thicknesses preferentially larger than 1 μm to allow for 100 nm deep indentation, thus avoiding misinterpretation due to surface and substrates effects; see Figure 4a. The scheme of this widespread thin film characterization method for the determination of elastic modulus and hardness is shown in Figure 5. A Berkovitch-type diamond nanoindenter is frequently used for such experiments, and a force–displacement curve is recorded during the indentation (loading) as well as during retraction (unloading) of the indenter from the sample. A depth-dependent nanoindenter surface contact function $A_{C}$ is needed to calculate the hardness and elastic modulus. Usually, a series of nanoindentations with varying depths are performed to decide the optimum indentation depth. The same setup can be used as a nanocompression experiment when running the indenter with a flat blunt tip; see Figure 6d. This allows for elastic modulus determination on compact and architectural structures; see Section 4.

The derivation of hardness and elastic modulus follows the procedure established by Oliver and Pharr [88]: with the contact area $A_{c}$ below the indenter tip at maximum force, the hardness can be calculated as

(10) $$H=F_{max}/A_{c}$$

The material’s reduced elastic modulus $E_{r}$ is

(11) $$E_{r}=\left(0.5\cdot \sqrt{\pi }\right)\cdot S/\sqrt{A_{c}}$$

where S is the slope indicated in Figure 5. The material’s elastic modulus E is obtained considering the indenter contribution $E_{i}$ via $E_{r}^{-1}=\left(1-\nu_{i}^{2}\right)E_{i}^{-1}+\left(1-\nu^{-1}\right)E^{-1}$, where ν is the Poisson ratio. Thus, hardness can be interpreted as the pressure the material can withstand below the contact area, i.e., its resistance against indentation.

#### 2.2.3. Plasticity and Fracture

Strength measurements subject the measured object to non-reversible shape changes in contrast to the elastic modulus measurements discussed previously. These non-reversible processes include plasticity and fracture. The three main methods to determine the onset of these non-reversible events are conceptually shown in Figure 6.

The fracture strength $\sigma_{f}$ of a pillar from a destructive bending experiment (Figure 6a) is related to the displacement $\delta_{f}$ at which fracture occurs [89]

(12) $$\sigma_{f}=\frac{3}{2}\frac{2r}{l^{2}}E\delta_{f}\;\mathrm{for}\;\mathrm{one}\;\mathrm{end}\;\mathrm{pinned}\;\mathrm{bending}.$$

Here, an ideal circular cross-section with a constant radius (*r*) over its entire length (*l*) is assumed. For tensile and compressive strain experiments (Figure 6b,c), the following relation derives directly from Hooke’s law of elasticity for objects with a constant cross-section along the load axis

(13) $$\sigma_{f}=E\frac{\delta_{f}}{l}\;\mathrm{for}\;\mathrm{uniaxial}\;\mathrm{tension}\;\mathrm{and}\;\mathrm{compression}.$$

In contrast to Equation (4)–(9) for the elastic modulus determination, Equation (12)and Equation (13) for the fracture strength determination do not require the experimental force *F* readout. Instead, the deflection $\delta_{f}$ suffices to determine the fracture strength, provided that *E* is known. However, the potential plastic yield onset, where deviation from Hooke’s linear elasticity relation between stress and strain happens, cannot be captured without a force sensor; see Figure 6d. The slope of the linear part of the curve in Figure 6d is proportional to the elastic modulus of the pillar. The compression experiment in Figure 6d was continued until fracture occurred. The related force can be directly read out and converted into strength by dividing with the circular cross-section of the pillar. As the pillar in Figure 6d has a hemispherical and slightly tapered top part, the calculated fracture force is slightly underestimated for small displacements within the top region, as the cross-section is smaller on top compared to the cylindrical region (the cross-section is used for the conversion of measured force into stress). Ideally, pillars should be straight and have a flat top, as shown in Figure 4f to facilitate the force (load) conversion into stress as well as to have a uniaxial stress distribution throughout the pillar.

**Figure 7:**
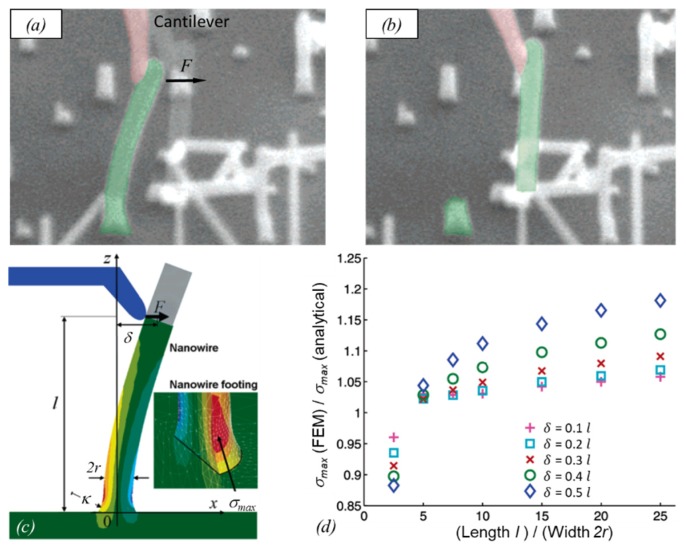
Bending fracture experiment with silicon nanowires inside an SEM. A cantilever (shaded red) bends a pillar (shaded green) laterally (**a**) until it finally breaks off (**b**). (**c**) Finite element simulations of the curved foot base of a pillar. Geometry of bending experiment and foot base with a curvature *κ* = 10^−2^nm^−1^. Largest tensile (red) and compressive (blue) stresses occur above the substrate–pillar interface. (**d**) Deviations due to curved foot base with respect to Equation (12), which assumes idealized uniform cylinder geometry. Modified from Hoffmann et al. [89].

Real bending fracture experiments are shown in Figure 7 before (Figure 7a) and after fracture (Figure 7b), where deviations from Equation (2) can occur due to the slightly broadened pillar footprints at the substrate interface, which is a typical feature of FIBID/FEBID pillars. Finite element studies by Hoffmann et al. [89], see Figure 7c, revealed that deviations from Equation (12) depend on the ratio l/(2r), where l is the length from pillar bottom to where the force is applied (not necessarily the pillar (beam) length) as well as on the amount of deflection δ. Figure 7d shows the normalized deviation between the analytical formula, assuming uniform circular cross-sections and finite element simulations, which take the broader base into account as well. The deviation ranges between 5% and 15% and increases with the length for a constant radius. 

#### 2.2.4. Density of FEBID and FIBID Nanostructures

**Figure 8:**
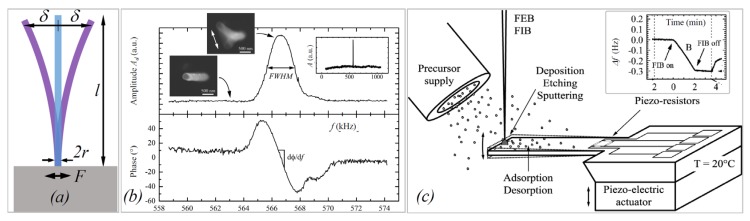
Density determination of FEBID/FIBID nanostructures. (**a**) Resonant vibration mode of single clamped pillars. The alternating excitation force can be applied via the pillar substrate through piezoactuators or via electrostatic forces surrounding the pillar. (**b**) Frequency sweeps of vibration amplitude (upper) and phase shift (lower) for a FEBID pillar across its fundamental vibration. The frequency inset shows the total 1 MHz scan and a single excitation, which is due to the uniformity of the deposited pillar. The SEM observation insets show the top view of the pillar in static and resonant mode. Modified from Friedli et al. [53]. (**c**) Mass measurement in the femtogram region via the frequency change of cantilevers during FEB or FIB deposition. The inset shows the shift in frequency during an FEBID deposition. Modified from Friedli et al. [46].

**Figure 9:**
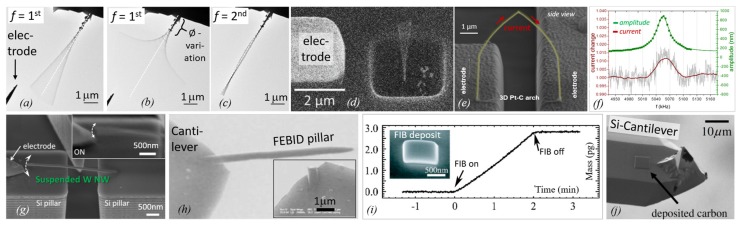
Selected overview of published methods for the density determination of nanostructures grown by FEBID and FIBID. (**a**–**c**) Pt-C FEBID pillar vibration with electric AC excitation inside a TEM. The first (a and b) and second fundamental resonances (c) can be seen. Note the large amplitude that the pillar can withstand as well as the diameter Ø variations. Modified from Hao et al. [90]. (**d**) SEM tilt view of an electrostatically AC excited Pt-C FEBID pillar vibration with integrated electrode design, modified from Arnold et al. [47]. (**e**) FEBID Pt-C bridge, which monitors vibrations in situ via the current read out shown in (**f**) modified from Arnold et al. [47]. (**g**) SEM tilt view of a laterally FIB grown pillar from W(CO)_6_ with integrated electrode. The inset shows the fundamental resonance. Modified from Cordoba et al. [51]. (**h**) Destructive mass measurements of FEBID pillars grown on a cantilever. Modified from Utke et al. [55]. **(i)** *In situ* mass measurement of a Pt-FIBID deposit deposited on an SEM integrated cantilever with piezoresistive frequency readout. Modified from Friedli et al. [46]. (**j**) Mass measurement of Ga-FIB grown carbon structure. The cantilever frequency was measured before and after the deposition. Modified from Kometani et al. [38].

The density of FEBID and FIBID material are typically determined by two approaches: (i) the resonant vibration of pillar structures or (ii) mass determination using femtogram sensitive balances, as sketched in Figure 8. The physics of free undamped flexural vibrations of pillars can be described by the Euler–Bernoulli equation [82]. For one end pinned (single clamped), uniform diameter, and uniform mass-distributed pillars, the resonance frequency of the first (or fundamental) vibration mode$\;f_{1}$, see Figure 8a,b, is [81,82]

(14) $$f_{1}=\frac{{\left(1.87510\right)}^{2}}{2\pi l^{2}}\sqrt{\frac{I}{A}}\sqrt{\frac{E}{\rho }}$$

where E is the elastic modulus, ρ is the density, I is the geometrical moment of inertia (second moment of area), A is the cross-sectional area, and l is the pillar length. The pre-factors of higher resonance modes were discussed by Friedli et al. [85]. In brief, cylindrical pillars are described by ${\left(I/A\right)}^{1/2}=r/2$; tubes follow ${\left(I/A\right)}^{1/2}=\sqrt{r_{i}^{2}+r_{o}^{2}}/2$, with subscripts i and o for inner and outer radius, respectively, and core–shell pillars with different material can be described by ${\left(EI/\rho A\right)}^{1/2}={\left[\left(E_{c}I_{c}+E_{s}I_{s}\right)/\left(\rho_{c}A_{c}+\rho_{s}A_{s}\right)\right]}^{1/2}$ [85] where the subscripts c and *s* denote core and shell materials, respectively, and ρA means the product of density and cross-sectional area. Several authors employed the pillar vibration method to determine the density by experiments; see Figure 9a–g. It is important to note is that Equation (14) contains the pillar’s elastic modulus and the density, which are two properties that are not known *a priori* for FEBID and FIBID materials. The elastic modulus needs to be determined beforehand by one of the approaches described in Section 2.2.1; then, the vibration experiments can be used to determine the unknown density of the pillar material [47].

An example of a resonance measurement is shown in Figure 8b. The vibration amplitude and the phase shift as a function of the excitation frequency of a piezoelectric actuator was detected by locking them to the electron beam signal [46]. Close to resonance, the pillar starts vibrating with a visible amplitude, which is reflected by a Gaussian peak in the amplitude versus frequency diagram (top). Alternatively, the excitation can be applied via electrostatic forces by using an electrode as was performed in the experiments shown in Figure 9a–g. Hao et al. [90] were able to excite also the second harmonic frequency $f_{2}$ (the pre-factor becomes ${\left(4.69409\right)}^{2}$ in Equation (14) in their FEBID pillars, as shown in Figure 9c. A peculiar feature of their experiment was that the pillar had a very non-uniform base part with large diameter variations (leading to non-uniform mass distribution), which probably explains their observation that the first fundamental vibration mode was excited at multiple frequencies (at $0.5f_{1},\;f_{1}$, and at $2f_{1}$ in our notation). Arnold et al. [47], see Figure 9d, carefully tuned FEBID conditions for straight uniform diameter pillars and suppressed fundamental resonance at multiple excitation frequencies. Córdoba et al. [51], see Figure 9g, deposited tungsten FIBID wires almost horizontally between two silicon blocks and excited them by an integrated electrode. The same setup was used for three-point bending experiments to determine the elastic modulus, see Figure 3d. Of note for AC excitation is that the electrostatic force contains a linear term with the excitation frequency and a quadratic term with the double excitation frequency. Hence, the correct assignment must be checked with double and half frequency. Of general note for density determination by vibration is that pillar non-uniformity in diameter and mass distribution as well as non-straight pillars will readily change the fundamental frequency defined in Equation (14) for the idealized pillar. Finite element analysis is necessary to cope with these deviations and to arrive at reliable density values [47,53]. 

Density can also be alternatively determined via its classical definition of mass divided by volume. The volume is relatively easily measured inside an SEM at various view angles. Figure 8c shows a cantilever-based mass sensor method that allows accessing the typical femto- and picogram weight range (10^−15^ g to 10^−12^ g) of nanostructures [46]. The principle is based on the sensitive change of frequency with changing mass, see inset, which is well known from quartz microbalances (working, however, only down to the nanogram range). This concept was used in the experiments shown in Figure 9h–j. Utke et al. [55] deposited freestanding pillars on silicon AFM cantilevers, see Figure 9h, and measured the cantilever frequency with pillar and after breaking off the pillar (in the AFM setup). Although being destructive, the advantage of this approach is that it cancels any unavoidable halo deposits by BSE and FSE adding extra mass. Friedli et al. [46] developed an SEM-integrated, piezoresistive cantilever-based in situ mass sensor, which they used to monitor mass uptake during FIBID and FEBID as well as mass reduction during FIB milling (used for in situ calibration). Figure 9i shows the mass increase of around 3 picograms during Ga-FIBID with Pt(CpMe)Me_3_ of a 1 μm × 1 μm footprint large pad. The root-mean-square noise in the measurement indicated a mass resolution of 10 femtograms. Kometani et al. [38] measured the frequency of a Si-cantilever before and after deposition in an AFM setup. Of note for cantilever experiments is that extra mass from thin halo depositions by BSE and FSE is included in the measurement. To avoid or minimize this systematic error, freestanding pillars or thin structures should be deposited at low electron energy. 

The mass-frequency response can also be applied to realize highly sensitive FEBID/FIBID-based sensor concepts, as demonstrated by Arnold et al. [47] The authors fabricated vertical pillars to determine the Young’s modulus and the material density via static and dynamic DC/AC experiments (see Figure 9d) together with finite element simulations in dependency on preparation parameters. After additional mechanical tuning via post-growth electron-beam curing (discussed in more detail later), they monitored the resonance frequency, while different gases were injected into the vacuum chamber. The latter led to resonance shifts, which is the basic concept for a sensor device. In the following, the authors changed the geometry from freestanding, vertical pillars into 3D arches, which bridged two electrodes. By monitoring the current through these bridges, the mechanical resonance (see Figure 9e,f) could be read out externally, which allowed the detection of surface adsorbed molecules down to (sub-)monolayer coverages. [47] For completion, Igaki et al. showed that the mass sensitivity of their high-aspect carbon FIBID pillar reached the 10 femtogram range [86], and Hao et al. [90] demonstrated the mass sensitivity of their Pt-C FEBID pillar to be 0.4 attograms. 

The fundamental resonance frequency can be also measured from natural thermal noise vibrations very much the same way as was discussed in Section 2.2.1. However, this method is limited to relatively large high aspect ratio resonating structures to be able to detect the minor thermally excited vibrations.

## 3. Mechanical Properties of FEBID and FIBID Materials

In this section, we will compare and discuss the available literature data concerning elastic moduli, hardness, yield strengths of plasticity and fracture, and densities. Most groups performed parameter scans during FEBID/FIBID to study their implications on mechanical properties. In the following graphs, we summarize the reported findings with a focus on the minimum and maximum values, as both are of interest for optimizing specific sensing applications. In addition, we discuss peculiarities that arise due to non-ideal shapes.

### 3.1. Elastic Modulus

The elastic moduli data available from the literature will be categorized into two sections discussing *carbon* and *metal–carbon* materials in Section 3.1.1 and Section 3.1.2, respectively. The different methods to determine the elastic modulus were explained in Section 2 and schematically summarized in Figure 3.

#### 3.1.1. Carbon FEBID and Carbon:Gallium FIBID Materials

**Figure 10:**
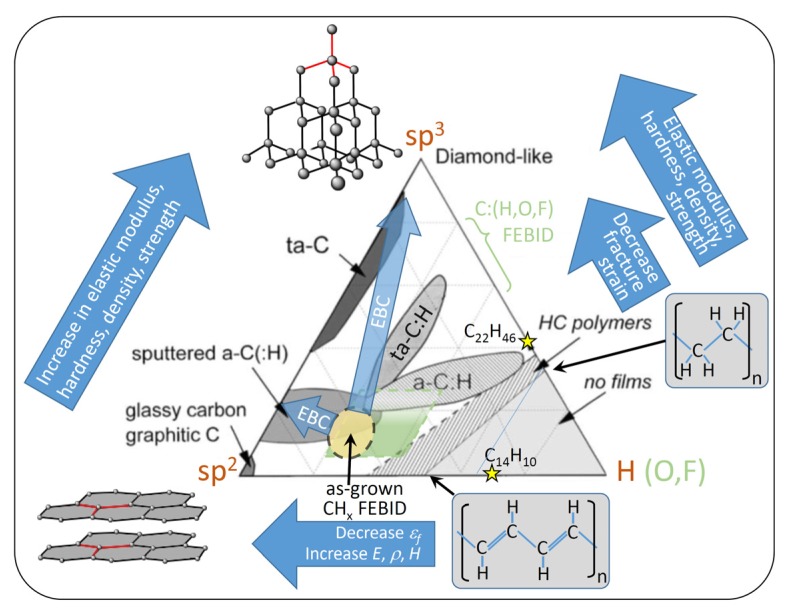
The ternary phase diagram of carbon materials with varying sp^2^, sp^3^, and hydrogen contents. Modified from Robertson [92]. The acronyms are amorphous carbon (a-C), tetrahedrally coordinated amorphous carbon (ta-C), amorphous hydrogenated carbon (a-C:H), and tetrahedrally coordinated amorphous hydrogenated carbon (ta-C:H). HC stands for hydrocarbon. Structural formulae are given for polyethylene and polyacetylene, sum formulae are given for paraffin and phenanthrene FEBID/FIBID precursors. Trends in mechanical properties are indicated by arrows as well as changes in composition during electron beam curing (EBC). The as-grown CH_x_-FEBID material space according to measurements from Bret et al. [93] and Ding et al. [45] is indicated (yellow shade) as well as the anticipated space for C:(H,O,F) FEBID (and FIBID) matrix material (green shade). No data are available for FIBID material.

Carbon material obtained from various thin film sputter and plasma-enhanced chemical vapor deposition processes is commonly categorized in the ternary phase diagram shown in Figure 10, as suggested by [91,92]. The sp^2^ and sp^3^ corners signify hybridization states of carbon corresponding to graphitic/glassy carbon and diamond, respectively, when no hydrogen is present. Moving to the hydrogen corner, carbon–hydrogen sp^3^ bonds will replaced the carbon–carbon sp^3^ bonds. Hence, the polymeric hydrocarbon (HC) contribution increases, where polyethylene (67 at.% H and 33 at.% sp^3^), polybutadiene (60 at.% H, sp^3^/sp^2^ = 1), and polyacetylene (50 at.% H and 50 at.% sp^3^) mark the hydrocarbons with highest hydrogen content being still able to form carbon networks. Beyond these hydrogen contents, no carbon film (or nanostructure growth) is possible, as the hydrocarbon units become volatile. The FEBID/FIBID precursors phenanthrene (C_14_H_10_) and paraffin (C_22_H_46_) were also indicated in the ternary phase diagram. The differing types of carbon film material obtained were branded according to their composition: (i) amorphous carbon (a-C) containing various disordered graphitic (sp^2^ bonds) material such as soot, chars, and glassy carbon, (ii) tetrahedrally coordinated amorphous carbon (ta-C), which is also termed diamond-like carbon with a three-dimensional carbon network via their four tetrahedrally arranged sp^3^ bonds, (iii) amorphous hydrogenated carbon (a-C:H), and (iv) tetrahedrally coordinated amorphous hydrogenated carbon (ta-C:H). The only study quantifying the hydrogen content in as-grown FEBID material from organic precursors from Bret et al. [93] found by Fourier transform infrared spectroscopy (FTIR) that the final carbon to hydrogen ratio in thick, about micron-sized deposits was C:H ≈ 4.5 (or CH_≈0.2_). With a FEB (25 keV, approximately 4 nA) using styrene (CH_2_=CH-C_6_H_5_) and other carboxylic acids, the hydrogen content in the deposits was about C:H = 9:2, where the hydrogen is mostly bound to the 5%–10% fraction of sp^3^-carbon atoms and less to the abundant sp^2^-carbon atoms (C=C bonds) conjugated with olefinic or aromatic units (having negligible H content). Of note is also that volatile residual gases in the chamber as well as very mobile surface contaminants may substantially contribute to the composition of the carbonaceous matrix with additional hydrogen [45] and oxygen [93]. Ding et al. [45] specified 80 to 90 at.% sp^2^ hybridized carbon in their as-grown FEBID material from paraffin. Combining the presently only available values of hydrogen and hybridization content for as-grown FEBID material gives the space highlighted in yellow in Figure 10. This space may serve as a rough indication and will change on the exposure parameters prevailing in the specific experiments, especially the irradiation dose, beam current, and electron energy. However, it is illustrative to see how the sp^2^, sp^3^, and hydrogen contributions of the original precursors change inside the deposit obtained by irradiation with primary electrons and ions, especially regarding the loss of hydrogen. 

Generalizing the hydrogen corner in Figure 10 to additional volatile elements, such as oxygen and halogens, as found in many organometallic FEBID and FIBID precursors and their corresponding deposited matrix materials as summarized in Table 1, allows situating them into the 60–80 at.% carbon composition band in the ternary phase diagram. The sp^2^/sp^3^ content distribution in the C:(H,OF) matrices was assumed to be similar to that measured for CH_x_ FEBID material; however, presently this assertion is of a hypothetical nature and awaiting experimental evidence. Furthermore, electron beam curing (EBC) stiffens as-grown FEBID deposits as it induces polymerization very similar to high-dose irradiated e-beam polymethethyl-methacrylate polymer resist [94]. This is indicated by the arrow in Figure 10 and would entail a further 3D carbon network formation with up to a 95% sp^3^ fraction increase as measured by Raman by Porrati et al. [95] and very probably hydrogen loss. 

**Figure 11:**
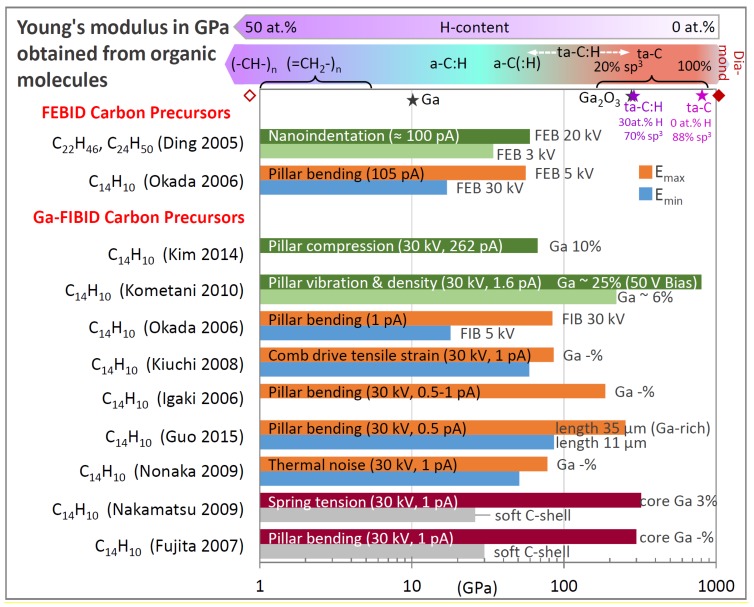
Summary of elastic moduli derived from as-grown FEBID and Ga-FIBID grown carbon materials; all using either paraffin (C_22_H_46_ and C_48_H_50_) or phenanthrene (C_14_H_10_). The vertical axis lists the precursor molecule used to grow the structure and the author reference. The measurement, deposition parameters (acceleration voltage, beam current), and the Ga implantation contents in at.% (when reported) for FIB deposits are indicated. On the top values and ranges of elastic moduli for polymers, various carbon types, diamond (E = 1143 GPa), gallium, and gallium oxide are shown for orientation. The trend in hydrogen content is indicated. Maximum and minimum values have specific legends when parameter studies were performed; otherwise, they show the experimental scatter. Note the logarithmic horizontal scale. Green colored bars represent measurements on homogeneous material, red-gray colored bars represent core–shell structures, and blue-orange bars made no core–shell distinction.

Depending on the hydrogen content and the sp^2^/sp^3^ hybridization state of the carbon, mechanical properties from soft-polymer and graphitic/glassy carbon up to diamond-like were reported; see the review of Robertson [92]. The situation is roughly indicated on the top part of Figure 11, keeping in mind that overlaps in the elastic moduli range of the various indicated carbon materials are likely as representatively shown for a ta-C:H carbon material with 70% sp^3^ and 30% H composition. Figure 11 contains a summary of all the measured FEBID and FIBID carbonaceous material elastic moduli in the literature. The various related measurement arrangements can be compared to in Figure 3 and Figure 4.

The FIBID deposition parameters of the structures investigated for the elastic moduli in Figure 11 were mostly done with phenanthrene at 30 keV acceleration voltage and beam currents of around 1 pA (except 262 pA for Kim et al. [41]) and allow for direct comparison. 

**Figure 12:**
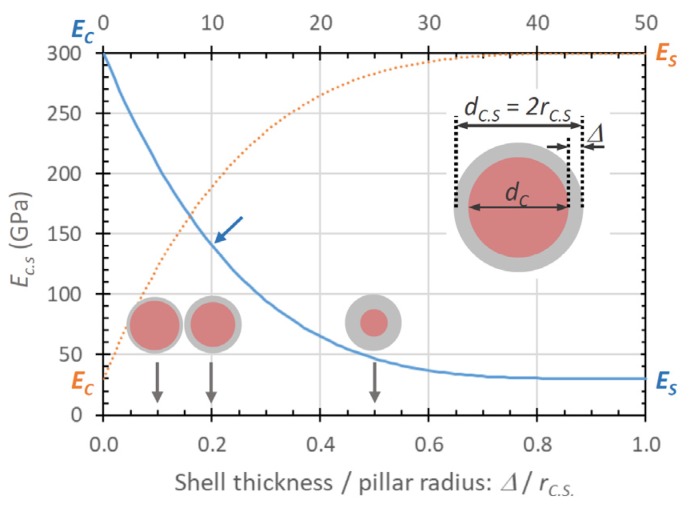
Core–shell pillar elastic modulus *E_C.S_* vs. shell thickness normalized to the core–shell pillar radius *r_C.S_* according to Equation (12) for the case of (i) stiff core/compliant shell (*E_C_* = 300 GPa/*E_S_* = 30 GPa) (blue line) as for pillar FEBID and FIBID, and (ii) compliant core/stiff shell (*E_C_* = 30 GPa/*E_S_* = 300 GPa) (orange dotted line) as may happen for e-beam curing. The total diameter 2*r_C.S_* was kept constant. The inset shows the cross-section geometry related to Equation (12). Furthermore, three core–shell cross-sections were visualized for indicated shell thicknesses to pillar radii. For discussion, see text.

Before entering into the details of Figure 11, we want to recall that FEBID and FIBID will result in core–shell pillar material when irradiating with a stationary focused beam, as illustrated and discussed in Figure 2. However, only Nakamatsu et al. [43] and Fujita et al. [44] measured this distinction explicitly for Ga-FIBID pillars and springs. Although core–shell formation is inherent to the carbon FEBID and FIBID process in stationary spot mode, moving the focused charged particle beam to grow larger structures than the spot diameter entails homogenization, and a spatially more uniform material distribution is obtained. This was most presumably the case for the nanoindentation experiments of Ding et al. [45] on FEBID carbon material and the nanocompression experiments of Kim et al. [41] on FIBID carbon material, as large micron-sized deposits had to be grown by scanning the focused electron/ion beam on this area. In addition, Kometani et al. [38] performed an experiment dedicated to attempt a radially uniform pillar distribution. All the other elastic moduli reported in Figure 11 represent values, which arise from pillars with unknown hard core–soft shell dimensions and do thus represent internal structure-related elastic moduli instead of characteristic properties of the constituent materials. The composed elastic modulus $E_{c.s\;}$of a core–shell pillar with an overall diameter diameter of $d_{c.s}$ can be calculated from the geometrical moments of inertia of the core and the shell, which sum up to the geometrical moment of inertia of the core–shell pillar, see for example [43,44]:

(15) $$E_{c.s}d_{c.s}^{4}=E_{c}d_{c}^{4}+E_{s}(d_{c.s}^{4}-d_{c}^{4})$$

where $E_{s}$, $d_{s}$ and $E_{c}$, $d_{c}$ are the elastic moduli and diameters of shell and core, respectively; see the inset in Figure 12 where Equation (15) is illustrated. As can be seen, thin shells, which have an elastic modulus of an order of magnitude smaller (or larger) than the core elastic modulus, can "hide" the core properties substantially. The numerical values for the blue line Figure 12 were chosen close to the measured core–shell pillar structure of Fujita et al. [44]. The blue arrow indicates their initially measured pillar elastic modulus *E_C.S_* = 140 GPa, before oxygen plasma etching the compliant carbon shell (30 GPa) from the stiff core (300 GPa). In other words, the co-deposited 10 nm thin compliant carbon shell made the elastic modulus of the pillar appear 50% less than the stiff core material contained therein.

In order to quantify the core and shell elastic moduli from Equation (15), four parameters out of five must be known. Nakamatsu et al. [43] (see Figure 4h) and Fujita et al. [44] removed the carbon shell from the gallium-rich carbon core material via oxygen plasma treatment and measured elastic moduli of around 300 GPa for the core material, as shown in Figure 11. Interestingly, this value corresponds to the elastic modulus of Ga_2_O_3_ (*E* = 250 to 290 GPa [96]) indicated in the top of Figure 11. This may hint to gallium oxide formation in addition to the soft carbon shell removal by the oxygen plasma, which would turn the rather soft gallium (E ≈ 10 GPa [97]) into a hard oxide. However, the carbon matrix is the constituent mechanical material given the low gallium content in the core material (< 10 at.% measured on the as-grown pillar) which is far below the percolation threshold of about 58 at.% of the gallium or gallium oxide, as discussed in Section 3.1.2. With the measured elastic modulus of about 300 GPa, this points to amorphous carbon with large sp^3^ hybridization content. The thickness of the removed soft shell carbon was specified at about 10 nm. The elastic modulus of the soft carbon shell was determined via Equation (15) to be in the range of 25–30 GPa [43,44]. 

With respect to the FEBID pillar core material, transmission electron microscopy diffraction analysis revealed amorphous carbon-containing nanocrystals [68]. The mechanical properties were not quantified, but the use of these core-sharpened pillars as nanoindenters into polymers showed an extraordinary endurance and no tip blunting. We now turn to homogeneous material. Kometani et al. [38] (Figure 4d) tried to minimize the formation of a core–shell structure by using a positive substrate bias of +50 V to keep the generated SE inside the growing pillar and thus minimize the dissociation of physisorbed phenanthrene molecules on the pillar side walls and the consecutive lateral growth of a carbon-rich shell. They showed that the amount of gallium increased from 6 at.% inside a confined core region at 0 V bias to 25 at.% more evenly distributed across a thicker cross-section at +50 V bias. The authors hypothesized that the bias voltage could have an influence on the collision cascade and caused FIB fluctuations (presumably focus and position) as possible causes. An extraordinary high elastic modulus of around 800 GPa was measured as well as a high density of 6.5 g/cm^3^; see section 3.2. As gallium is very compliant (E= 10 GPa), it may be hypothesized that the carbon matrix must have changed considerably from sp^2^ to sp^3^ hybridization and low hydrogen content to compensate this compliant contribution. However, no such material characterization was shown by the authors, so the role of the secondary electron confinement for the carbon material of the extraordinary stiff pillar material still remains elusive. Kim et al. [41] deposited 5 × 5 × 5 μm^3^ carbon cubes by Ga-FIB (30 kV, 262 pA) using phenenthrene; see Figure 4f. The carbon material contained 10 at.% of Ga, 90 at.% carbon, and 0.3 at.% oxygen; hydrogen was not measured. The elastic modulus and the compressive fracture strength were measured as 67 GPa and 8 GPa, respectively. The elastic modulus is very close to the highest value found for FEBID deposits from Ding et al. [45], which is also shown in Figure 11. They deposited with an FEB (3, 12, 20 keV, ~100 pA) and paraffin precursors (C_22_H_46_, C_24_H_50_, C_24_D_50_) carbon material with roughly 15 × 15 × 0.25 μm^3^ volume, which they used for nanoindentations experiments. The material was extensively characterized: the sp^2^/(sp^2^+sp^3^) fraction was 0.92 (3 keV), 0.87 (12 keV), and 0.83 (20 keV); it was determined by electron energy loss spectroscopy (EELS) and hydrogen (deuterium) was qualitatively detected by secondary ion mass spectrometry (SIMS). The authors concluded that the FEBID material was amorphous, had more sp^2^-bonded C than sp^3^-bonded C, and contained some H. The hardness and elastic modulus values of the FEBID material obtained for 3, 12, and 20 kV were measured as 3.6 ± 0.3, 4.0 ± 0.2, and 4.4 ± 0.2 GPa, and 34.3 ± 3.4, 46.3 ± 2.3, and 59.5 ± 2.5 GPa, respectively, and they follow the measured trend of elastic modulus (and hardness) increase with increasing sp^3^ fraction due to the increasing incident electron energy. According to Figure 10, the corresponding carbon material is slightly hydrogenated amorphous carbon a-C(:H).

We are turning now to studies that give average elastic moduli instead of making a distinction between possible core and shell structure properties. Of note is the comparative FEBID/FIBID study from Okada et al. [36] in dependency on the acceleration voltage. Both are shown in Figure 11 and reflect an opposite trend: while the stiffer FEBID carbon material (56 GPa) was obtained at 5 kV, the stiffer Ga FIBID material (84 GPa) was found for 30 keV FIBID. The differences for electron and ion interactions in pillars as a function of incident energy were already shown in Figure 2a,c and discussed in the related paragraph. Accordingly, one can attribute the increasing stiffness with increasing ion energies during FIBID to the increasing energy dissipation and the Ga-rich core, which increases in diameter. While the latter evidently results in stiffer pillars, the role of an increased ion energy for material stiffening is not fully comprehensive, as the amorphization (collision cascade) also increases, which in turn would detriment stiff sp^3^ tetragonal-coordinated carbon aggregations. However, the generated heat in the pillar apex would favor re-ordering processes of amorphous material due to improved mobility at the atomistic scale. For FEBID, it was shown in Section 2.1 that the implanted energy is not only a function of primary electron energies but also depends on the deposit size. For situations where the excitation volume is much smaller than the deposit volume, the dissipated energy would scale with the primary energy. This is the trend observed via indentation experiments by Ding et al. [45], as shown in Figure 11 and Figure 4a. From the previous discussion of Figure 2, we saw that this trend reverses for small diameter pillars, which was found by Osaka et al. [36] in his experiments. 

For the remaining experiments giving averaged elastic moduli, there are >200 GPa values reported for long horizontal pillars from Guo et al. [37] (Figure 4c) and for bars with rectangular cross-sections from Igaki et al. [86] (necessitating scanning of the Ga-FIB and thus Ga-homogenization as found for Kometani et al. [38]). These seem to be related to richer gallium contents; however, they were only qualitatively mentioned. The same comments apply as discussed above for the results from Kometani et al. [38]. The experiments of Nonaka et al. [42]], see Figure 4g, and Kiuchi et al. [39], see Figure 4e, gave maximum elastic moduli of 70–90 GPa. They were using different techniques; Kiuchi et al. [39] deposited horizontal pillars onto a comb drive for tensile experiments and Nonaka et al. [42] used the thermal vibration technique to determine the elastic modulus. Without any chemical characterization of the carbon material, it is difficult to judge if the modulus scatter corresponds to the material composition (sp^2^/sp^3^, hydrogen content) or to the measurement technique involved.

When generally comparing the as-grown material of FEBID against Ga-FIBID, it can be noted from Figure 11 that FIBID gallium-contaminated carbon material C:Ga material populates a rather large range from about 70 to 800 GPa (considering the maximum values). The two available carbon FEBID materials are close to each other around 70 GPa with their maximum measured values and thus comparable to the lower FIBID range. The high-end range of FIBID material elastic moduli is getting close to pure diamond with E = 1143 GPa [98]; however, this material corresponds to Ga-enriched carbon pillars. The implantation of gallium during Ga-ion FIBID is intrinsic to the process and cannot be fully avoided. Yet, it can be modulated by the deposition rate. Supplying an intense precursor molecule flux from several gas injection systems will enable tuning of the Ga-ion dose as the increased deposition rate requires less time for pillars reaching a given length and the overall implanted Ga content will diminish. What seems most important to turn out is the spatial homogenization of the Ga content inside the pillar. Interestingly, the elastic modulus of the compliant carbon shell in FIBID core–shell treated pillars is around 30 GPa, which corresponds to the minimum range of elastic moduli of around 20–40 GPa measured in FEBID. Assuming that lateral growth in FIBID pillars is due to the secondary electron generation by the incident ions and the collision cascade, this would mean that the same carbon deposition mechanisms take place as in FEBID (neglecting the incident keV electrons), and thus roughly the same material properties can be expected.

For completeness, we also cite a very high elastic modulus for FEBID derived from vibration measurements using assumed values for the material density. Jaenchen et al. [99] measured 540 GPa for tapered FEBID carbon tips grown from residual surface and gas phase hydrocarbons. However, we would like to mention that these values would need consolidation by independent density measurements. 

As an intermediate section conclusion for deposition with hydrocarbon precursors, FIBID C:Ga high aspect ratio pillars reach elastic moduli in the 200 to 800 GPa range. This modulus range is found for tetragonally coordinated amorphous carbon ta-C as well as for hydrogenated tetragonally coordinated amorphous carbon ta-C:H at the low end of the modulus range. This points to a larger efficiency of Ga ions to form sp^3^ carbon bonds, despite the amorphization in the collision cascade. Most probably, temperature effects and secondary electron generation help to reduce the hydrogen content and to form a diamond-like carbon matrix in pillars. The limited number of experiments on voluminous low aspect ratio FIBID and FEBID structures show comparable elastic moduli with maximum 70 GPa. This corresponds to slightly hydrogenated amorphous carbon material a-C(:H) having a relatively large graphitic content. Furthermore, stationary spot mode deposition results in stiff carbon core and compliant carbon shell pillar structures. Consequently, attention should be taken if papers distinguish between the core and shell mechanical properties or give overall efficient elastic moduli, seeEquation (15). The chemical characterization with respect to the ternary sp^2^-sp^3^-H phase diagram is missing presently and would help to gain deeper understanding on the influence of deposition parameters. The role of implanted gallium and its uniform versus non-uniform distribution is still elusive. For the moment, we were treating the role of Ga as negligible in the carbon matrix due to its small elastic modulus and were not discussing FIBID carbon deposit properties in the following metal carbon materials section. 

#### 3.1.2. Metal–Carbon Materials

**Figure 13:**
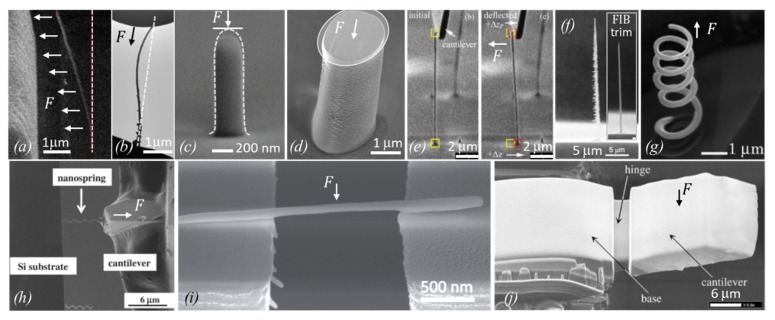
Overview of published metal–carbon deposit shapes and methods to determine their elastic moduli. The force *F* acting on the structure is indicated as well as the initial shape of the structure (dashed line). (**a**) Pt-C FEBID pillar bent within an electrostatic field toward the electrode, modified from Arnold et al. [47]. (**b**) Buckled Pt-C pillar between an actuator (top) and substrate (bottom). The initial shape was straight as indicated, modified from Hao et al. [90]. (**c**) Pt-C FEBID pillar nanocompression by a flat diamond punch (not shown), modified from Lewis et al. [48]. (**d**) FEBID Co-C pillar with Ga–FIB milled flat top for compression experiments. (**e**) Cu–C pillar bending with cantilever and pillar base image tracing, modified from Friedli et al. [53]. (**f**) FIB pillar from W(CO)_6_ giving large lateral spike shapes (left), which were FIB-milled for better shape assignment in bending experiments, modified from Ishida et al. [100]. (**g**) FIB grown helix with W(CO)_6_ and C_14_H_10_ for tensile strain experiments, modified from Nakamatsu et al. [43]. (**h**) Tensile strain experiment with Fe-C FIB helix fixed between a cantilever and substrate, modified from Nakai et al. [59]. (**i**) FIB W-C horizontal rod three-point bending experiments, modified from Córdoba et al. [51]. (**j**) FIB grown silicon oxide structure with FIB-milled hinge geometry for bending experiments, modified from Reyntjens et al. [62].

As for the carbon structures discussed in the previous section, different approaches were applied to determine the elastic moduli for nanogranular metal–carbon composite materials, which are further denoted as nanogranular materials for simplicity. The approaches from different groups shown Figure 13 resemble partly the ones already discussed in the previous section.

Arnold et al. [47], see Figure 13a, used an electrostatic field to bend the Pt-C FEBID pillars within toward the electrode and calculated the elastic modulus via finite element simulation of the spatially distributed force field. Hao et al. [90] is the only reference using Eulerian pillar buckling for modulus determination. Although the method seems simple, as shown in Figure 13b, it is not easy to judge how the top load is fixing the pillar. Cases of free-rotating, pinned, and hinged can be distinguished and change the pre-factor K in Equation (7) from 0.5 to 1, which seems the largest error in this type of measurement. Figure 13c shows Pt-C FEBID pillar nanocompression by a flat diamond punch from Lewis et al. [48]. The dotted line was the initial pillar shape. The smaller shape of the pillar after nanocompression witnesses plastic contributions. Figure 13d shows a thick FEBID Co-C pillar which was flat top milled by Ga-FIB to obtain uniform load distribution along the pillar cross-section during nanocompression. Typically, FEBID and FIBID do not naturally result in flat top pillars, as seen in Figure 13c. The deposition parameters would need to be very carefully tuned [73,101]. However, if thick pillars can be grown large enough, then cutting away the round top by Ga-FIB is an option as far as no changes in the pillar material are induced. Figure 13e shows the bending of high aspect ratio Cu-C pillars with a cantilever of known force constant. Image tracing of the pillar base and top allowed Friedli et al. [53] to calculate sub-pixel resolution in the readout of the deflection; see the yellow (reference) and red (deflection) squares. Figure 13f is informative with respect to as-grown Ga-FIBID pillar shapes obtained by Ishida et al. [100]. Their FIB pillars from W(CO)_6_ resulted in large lateral spike shapes (left), which were FIB-milled for better shape assignment in their bending experiments. It is likely that this procedure gives a thin shell of implanted Ga, which may oxidize with time from the oxygen inside the deposit. As Ga_2_O_3_ has a modulus of 290 GPa [96], it may influence the measured pillar modulus; compare to Figure 12. Nakamatsu et al. [43] grew FIB helices with combined W(CO)_6_ and C_14_H_10_ gas injection, see Figure 13g. They fixed a cantilever tip at the helix end and pulled the helix for tensile strain experiments. It shows the high capability of 3D control for the FIBID process at the nanometer scale as well as the challenges involved in obtaining such structures in an ideal shape for the mechanical measurements. The actual tensile strain experiment is shown in Figure 13h with an Fe-C FIB helix fixed between a cantilever and substrate from Nakai et al. [59]. Córdoba et al. [51] succeeded in growing a horizontal rod from W(CO)_6_ along two pre-etched silicon pillars for three-point bending experiments; see Figure 13h. The fixation of both rod ends is essential to apply the formula in Equation (7); however, it may not be easy to assure. In Figure 13j, Reyntjens et al. [62] fabricated via Ga-FIBID a free-standing silicon oxide cantilever structure and milled a small groove (hinge) into it. Together with finite element simulations, they calculated the elastic modulus of the deposit. During milling, additional Ga ions will be implanted below the silicon oxide material surface and may change the modulus much the same way as for the core–shell structures discussed in Figure 12.

**Figure 14:**
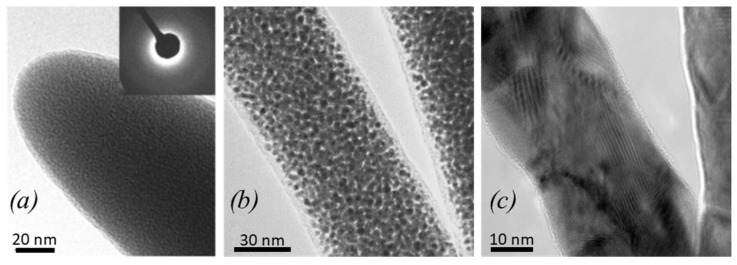
TEM images of FEBID nanopillars using differing precursors and post-treatments. (**a**) Amorphous dielectric Si-O-C tip from tetraethoxysilane (TEOS), fabricated at 25 keV/100 pA. (**b**) shows an Au-C nanopillar shaft fabricated at 30 keV/21 pA using Me_2_Au(acac) precursor, revealing 3–5 nm Au nanograins (dark), which are embedded in a carbon matrix (bright). (**c**) shows the same pillar after full purification using 5 keV/1.2 nA in 10 Pa H_2_O environments, where the highly crystalline Au character becomes evident. Of particular relevance is the widely maintained morphology, although diameters are strongly reduced (see scale bars). The remaining surface contamination by carbon due to the imaging is only about 1 nm, which becomes essential for plasmonic applications [75].

Before discussing the mechanical properties of FEBID and FIBID nanostructures obtained from metalorganic precursors, we would like to point out some differences, which occur in the material in relation to the use of organic precursors. Due to the presence of a metal element in the precursor, several internal structures within deposits can occur, which range from amorphous and nanogranular to crystalline depending on the final content and oxidation state of metal in the deposit. Figure 14 illustrates these types of internal structures. The embedding matrix composition depends on the precursor molecule and the deposition parameters but often contains all ligand elements; see also Table 1 in Section 2.1.

**Figure 15:**
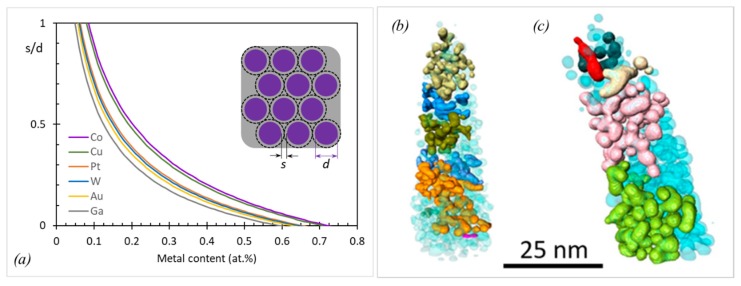
(**a**) The average nanoparticle distance *s* normalized to their size *d* versus the metal content for five metals. For s/d=0, the full percolation metal threshold is reached. The inset shows the corresponding idealized close packed geometry of metal nanoparticles (purple) with a carbon shell (gray, dotted line). The carbon matrix density was set to 2 g/cm^3^. (**b**) and (**c**) show 3D reconstructions from Pt-C nanopillars, which were derived from high-resolution TEM tomography. (**b**) shows the as-grown FEBID with 30 keV/21 pA, with large Pt nanocrystals becoming evident and revealing slightly percolated characteristics, as indicated by the different colors. After high-dose e-beam curing at 30 keV/150 pA, the grain growth (average by 25 rel.%) is clearly evident, while the partial percolation characteristics remain, as shown by the different colors in (**c**). Images have been adapted from reference [102].

Metal-based properties (electrical conductivity, magnetic moment, etc.) will determine the pillar function when the nanogranular material will start to percolate. Assuming the nanogranular material to be ideally composed of monodisperse-sized metal sphere–carbon shell units as shown in the inset of Figure 15, the percolation metal content can be estimated by [11]

(16) $$\frac{s}{d}={\left\{\frac{1}{P}\left(\frac{1-x}{x}\cdot \frac{\rho_{np}}{\rho_{m}}\cdot \frac{M_{m}}{M_{np}}\right)+1\right\}}^{1/3}-1$$

where s/d is the ratio of the average nanoparticle spacing (two times the thickness of the carbon shell) to the nanoparticle diameter and thus a measure of percolation, x is the metal atomic ratio in the metal matrix material, and ρ and M are the density and the molar mass of the nanoparticle (np) and the matrix (m), respectively. The packing factor P depends on the type of packing and is P≈1.35 for close packing geometries. Figure 15a shows that percolation metal contents in a 2 g/cm^3^ dense carbon matrix are situated around 60 at.% (Ga), to 65 at.% (Au, W, Pt) to 75 at.% (Co, Cu). For any metal matrix combination, the percolation metal threshold derived from Equation (15) is given by $x_{P}={\left(1+P\rho_{m}M_{np}/\rho_{np}M_{m}\right)}^{-1}$. However, this model is idealized with respect to homogeneity, and in real FEBID/FIBID materials, more or less narrow size and space distributions of nanocrystals inside the matrix can occur, as seen in the as-grown Pt-C deposit in Figure 15b as well as after e-beam curing in Figure 15c. However, with increasing metal content, these inhomogeneities will naturally decrease.

As for the carbon pillars discussed in Section 2.1., core–shell structures may also arise for pillar deposition in metal FEBID [103] and FIBID, and care should be taken to tune deposition conditions and gas delivery for homogeneous pillar material deposition or to carefully investigate the internal structure for proper interpretation of the results, especially when mechanical properties are studied. Due to the nanometer scale size of FEBID and FIBID structures, the pillar characterization with respect to radial and axial uniformity is very challenging and requires analysis methods with sophisticated preparation, such as transmission electron microscopy ([102], see Figure 14), FIB cross-sectioning [103], and an atom probe [104]. 

**Figure 16:**
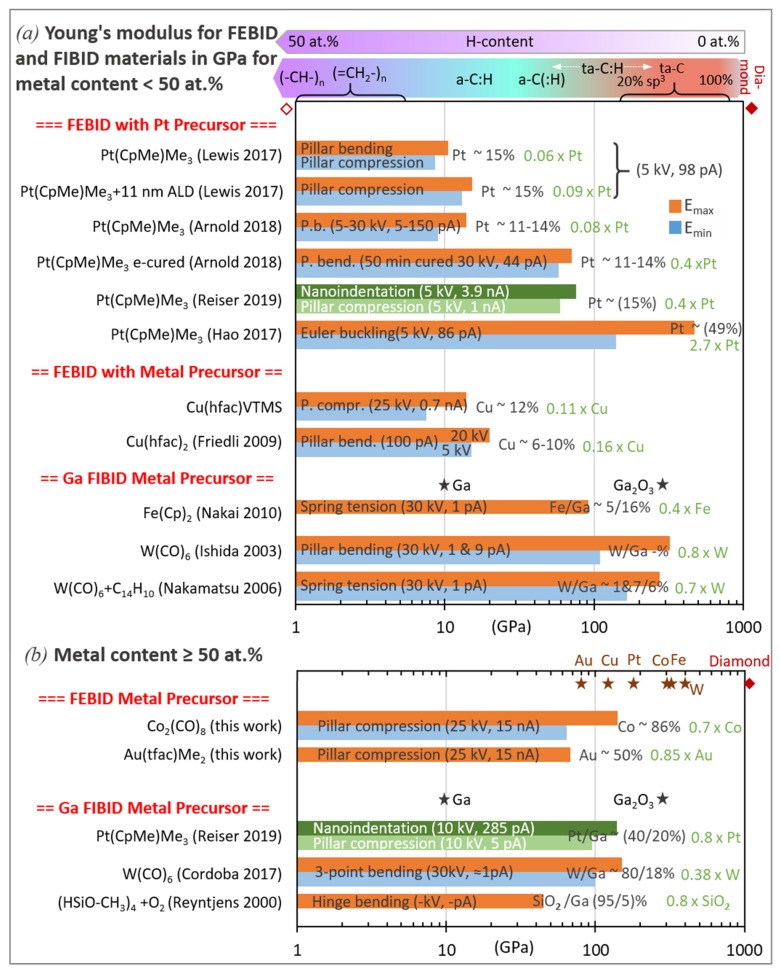
Summary of elastic moduli measurements of FEBID and FIBID-grown metal–carbon materials. The vertical axis lists the precursor molecule employed to grow the structure together with the first author. Bulk elastic moduli for metals, gallium, gallium oxide, and diamond are shown for orientation. The data point entries give the corresponding elastic modulus ratios of deposit to bulk metal. Metal contents are in at.% (parenthesized values were inferred from separate literature). The measurement methods and deposition conditions (acceleration voltage, beam current) are shown inside the bars. Maximum and minimum values have specific legends when parameter studies were performed; otherwise, they show the experimental scatter. Green shades signify measurements on uniform material that has no potential core–shell structure hidden. **(a)** Deposits with metal content below percolation threshold and **(b)** deposit material with metal content >50 at.% and percolation threshold. Note the logarithmic horizontal scale.

Data of the elastic moduli found in the literature are summarized in Figure 16 together with the metal content, focused charged particle beam deposition parameters, and type of measurement. For comparison, bulk metal elastic moduli are shown in the top line of the diagram: Pt, 174 GPa; W, 396 GPa; Cu, 125 GPa; Fe, 216.8 GPa; Co, 203.8 GPa; Au, 80.2 GPa [105]; Ga, 9.8 GPa [97]; Ga_2_O_3_, 290 GPa [96]. 

For all metal–carbon compositions, the fraction of each measured deposit modulus with respect to the corresponding bulk metal modulus is given in the legend (green text). The lower metal content materials (Figure 16a) can be rather compared to the carbon matrix elastic moduli, which were discussed in the previous section. Only few materials have metal contents above 50 at.%: 86 at.% cobalt FEBID material (this work) and 80 at.% tungsten FIBID material [51] being larger than the percolation threshold together with the 95 at.% SiO_2_ FIBID sample. The Au-C and Pt-C [49,90] materials have still metal contents of around 50 at.%. These high metal content materials were grouped in Figure 16b. 

We discuss the materials according their grouping in Figure 16 in the following sections.

##### Platinum FEBID and FIBID Material

This material is typically obtained from the trimethyl-Pt-cyclopentadienylmethyl precursor available as standard gas injection system add-on from most SEM manufacturers; see Table 1. Most FEBID experiments report 10 to 15 at.% platinum content (excluding hydrogen) in the deposit, which was supported from surface science studies, identifying the loss of one CH_3_ group as the dominating electron dissociation reaction leading to deposition. For reasons discussed in relation to Table 1 in Section 2.1., there are presently no studies that attempted to quantify the hydrogen content in the deposit. Most probably the stoichiometric carbon:hydrogen ratio of C : H = 9:16 in the precursor compound, i.e., about 2 hydrogen atoms per carbon, will decrease upon electron irradiation and result in polymerized carbon networks with hydrogen content varying according to the specific irradiation conditions. The mechanical properties of such carbonaceous matrix material were discussed in Section 3.1.1.

From Euler buckling experiments, see Figure 13b, Hao et al. [90] report a 2.7 times larger elastic modulus than bulk platinum for FEBID material with an atomic ratio of Pt:C ≈ 1:1. This metal content seems overestimated for the notorious Pt-Cp precursor, and indeed, the Pt-content was only inferred from mechanical properties and not measured directly. In addition, their tapered pillar structure (large lateral deposits at the pillar base, see Figure 13b) may give rise to over interpreted values when applied to ideal cylinder geometry. According to Figure 15a, the percolation for platinum starts around 65 at.%, so that one can conclude that the carbon matrix and not the metal dominates the mechanical properties in this material; as the measured elastic modulus E is 470 GPa, the carbon matrix falls into the ta-C category with large sp^3^ and low H content, as discussed in Figure 11. This modulus value is very high compared to the other as-grown Pt-C FEBID material reported so far (Figure 16), but it is also very high compared to the FEBID carbon material summarized in Figure 11. Interestingly, Hao et al.’s work is the only one that used 300 keV electrons during the mechanical measurements inside a TEM. Such high energy electrons can easily knock-out light elements such as hydrogen, which was still contained in the as-grown (5 keV, 89 pA) material. Thus, we hypothesize that during their experiments inside the transmission electron microscope, e-beam curing occurred simply during observation, leading to a densified, sp^3^-reticulated, low hydrogen carbon network material, as was also reported by Arnold et al. [47] and Porrati et al. [95]. The work by Lewis et al. [48] resulted in 15 at.% Pt FEBID content and elastic moduli of 8.6–10.5 GPa for the as-grown material determined by two different methods (pillar bending and compression), see also Figure 13c which assures the viability of the measurement approaches. Coating the pillars with an 11 nm thick and stiff Al_2_O_3_ atomic layer deposition (ALD) film at 100 °C increased the moduli slightly to 15.6 GPa. The elastic modulus of ALD Al_2_O_3_ films grown at 100 °C is about 150 GPa. Entering the values into Equation (15) gives a core–shell structure modulus of about 30 GPa, which would be expected. The discrepancy may arise due to the pillar nanocompression method exclusively applied for the coated structure. Arnold et al. [47], see also Figure 13a, achieved for similar 15 at.% Pt content material also similar elastic moduli as Lewis et al. [48] for the as-grown FEBID material in the range of 9–13 GPa. Furthermore, Arnold et al. [47] demonstrated a powerful approach of post-growth e-beam curing (EBC) to tune the mechanical properties enabling precise tuning of the elastic modulus. After FEBID, Pt-C nanopillars were brought into mechanical resonance via AC fields to enable homogenous EBC (post-growth exposure of the as-grown pillar to 30 keV electrons without gas injection during 50 min) at both sides of the pillar. The pillar diameter changed only slightly by 2 nm, the composition stayed the same, while Pt-grain growth was observed. Based on the gradual resonance shift, the elastic modulus could be derived and coupled to the exposed EBC dose, which revealed (i) an increasing elastic modulus from 14 GPa (as deposited) to 70 GPa and (ii) a reduction of intrinsic energy losses during oscillation, as reflected by a Q^−1^ decay from 9.8 × 10^−3^ to 6.7 × 10^−3^. The authors attributed the increasing stiffness again to the formation of sp^2^ carbon bonds and eventually to sp^3^ carbon networks. Their study also points out that the Young’s modulus did not show a saturation behavior at 70 GPa, which means that even higher stiffness for Pt-C nanopillars are very likely. Thus, the tuning of mechanical properties of carbonaceous FEBID material is easily achievable and very useful for potential nanomechanical applications. It can be understood in a way that EBC will transform the carbonaceous matrix still rich in hydrogen into material with less volatile elements with curing time and increasing stiffness by the formation of sp^2^ and sp^3^ carbon bonds; see also the ternary phase diagram in Figure 10. The study of Reiser et al. [49] used two methods to determine the elastic modulus independently and revealed 60 to 75 GPa for nanoindentations on about 10 × 10 × 0.5 m^3^ pads and 1 × 1 × 2 m^3^-sized pillar compression. The currents used were 3.6 nA and 1 nA, respectively, and they accumulate a relatively large dose in the growing deposit volume, which leads to the simultaneous e-beam curing of the growing structure. The authors argued that this is the cause for the relatively large elastic moduli comparable in value to Arnold et al.’s [47] e-beam curing results. It is of note that for the experiments of Reiser et al. [49], homogeneous material can be expected (no core–shell pillar structures), as large area deposits in the micrometer range were measured (which necessitated e-beam scanning), in contrast to the beforehand discussed pillar geometries in this section. Summarizing the FEBID Pt-C results presented above (i), the carbon matrix dominates the elastic modulus, values from 0.06× to 2.7× of the platinum modulus were obtained, and (ii) e-beam curing offers a convenient possibility to tune the properties very precisely from compliant (approximately 10 GPa) to stiff (450 GPa) according to the application needs.

##### W–, Co–, Au–, and Cu–Carbon FEBID Materials: 

According to Figure 15, only the cobalt–carbon material is above the percolation threshold, as it has a metal content of 86 at.%. This is reflected in an elastic modulus that is 70% close to pure bulk cobalt. In addition, the gold–carbon material features a modulus that is 85% close to bulk gold, although the gold content with around 50 at.% is slightly below the percolation limit of around 60 at.%. Of note is that both materials were grown with relatively large beam currents of 15 nA in comparison to the typical 100 pA deposition current. In contrast, the low-metal content FEBID copper–carbon materials feature elastic moduli of around 11%–16% of the corresponding bulk metal moduli, which is due to metal contents below the percolation limit. Consequently, the elastic moduli are dominated by the state of the carbonaceous matrix, which comprises its composition as well as its hybridization state. According to Table 1, the carbonaceous matrix of the copper FEBID materials contains several volatile elements (hydrogen, fluorine, and oxygen), which are very likely forming soft polymer-like hydro- and fluorocarbons with oxygen groups. As for the amorphous hydrogenated carbon matrix obtained from the platinum precursor discussed previously, also the mechanical properties of such matrix material can be substantially tuned under electron radiation, i.e., e-beam curing [47]. Evidence for such behavior was found by Friedli et al. [53] and Utke et al. [55] with respect to matrix densification upon electron dose variations (see Section 3.2), but it was not fully exploited for elastic modulus tuning. For the FEBID tungsten material, values on composition are missing, and we speculate that the tungsten amount is below the percolation limit so that the carbonaceous matrix will determine the relatively low elastic modulus with respect to pure tungsten. The homogeneity of the pillar structures in this section was not investigated so that specific core–shell features cannot be ruled out. 

##### Pt–Carbon, Fe–Carbon, W–Carbon, and Si–Carbon FIBID Materials

Investigations were performed on homogeneous material by Reiser et al. [49] for Pt-C containing about 40 at.% Pt and 20 at.% Ga (this composition was taken from Friedli et al.’s Pt-C FIBID experiments [46]). Thus, the total metal content adds to about 60 at.%, which is around the percolation threshold concentration for both metals; see Figure 15. Then, the atomic percentage weighted elastic modulus average of Pt and Ga is about 125 GPa, which fits relatively well to the measured values in Figure 16. The remaining 40 at.% hydrogenated carbon matrix may also contribute to stabilize the percolated metal to the overall elastic modulus. Of note is that two different methods (pillar compression and nanoindentations) gave approximately similar results on different structures: 90 GPa versus 120 GPa for 1 × 1 × 2 m^3^ sized pillar versus 10 × 10 × 0.5 m^3^ pads, respectively. This hints to good uniform material deposition with only subtle differences in carbon matrix hybridization and hydrogen content. FIBID of SiO_2_ is also known to result in rather pure material; see Table 1. Not surprisingly, the elastic modulus measured for this material by Reyntjens et al. [62] reaches values of 80% of the bulk silicon oxide. The 20% weakening may be attributed to the incorporation of compliant gallium and non-stoichiometric SiO_x_ material. As the FIB was scanned in this experiment to create a large volume structure, no core–shell features were expected. With respect to W-FIBID, three references were found. Córdoba et al. [51] showed that horizontally grown pillars had a core–shell structure containing a roughly 80 at.% W (nanocrystalline) and 18 at.% Ga core and a nanocrystalline and roughly 6 nm thick 95 at.% W shell with some carbon and oxygen. Despite the high tungsten core content (above the percolation threshold, compare Figure 15), the core–shell elastic modulus was only about 40% of bulk tungsten. The authors concluded that the outer shell could be pure tungsten with the bulk elastic modulus of 521 GPa, which embeds a compliant gallium contaminated tungsten core. However, estimating their W:Ga core elastic modulus with Equation (15) gives a very small value of about 20 GPa, which seems to contradict their observations of the nanocrystalline nature of the core and also in part their fracture yield results discussed in Section 3.3. Therefore, we did not include core and shell elastic moduli in Figure 16, but we also would like to highlight that the task is not easy; stiff shells will probably by nature complicate any attempt of their removal from the compliant core and thus prohibit the successful plasma removal approach used for compliant carbon shells around stiff carbon cores [43,44,68]. The W-FIBID work of Nakamatsu et al. [106] shows larger elastic moduli of about 70% of bulk tungsten although, only 1–7 at.% of tungsten were in the material (note that the authors used phenanthrene as a second gas to mix with W(CO)_6_). This highlights again that the carbon matrix is contributing largely to the mechanical behavior and reached about 300 GPa, which is typical for core carbon material; see Figure 11. Similar arguments apply for the Fe-FIBID of Nakai et al. [59], where the iron content is far below percolation. Their elastic modulus of around 90 GPa falls into the lower range of measured FIBID material elastic moduli; compare to Figure 11. 

**Figure 17:**
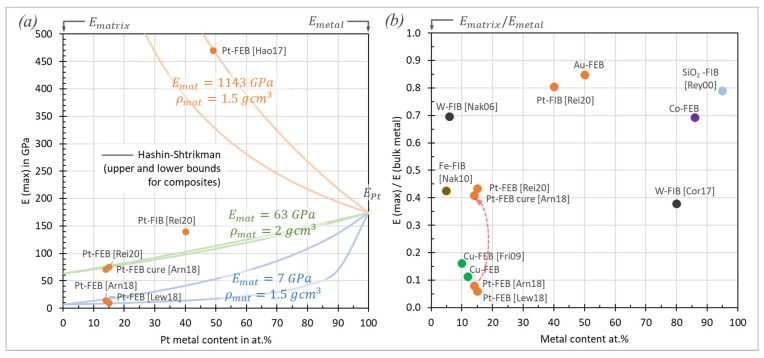
Elastic moduli of FEBID and FIBID material vs. reported metal content. (**a**) For Pt–carbon deposits. Composite elastic moduli according to the Hashin–Shtrikman upper and lower bounds are shown as lines. The corresponding numerical values represent fits to the data points. (**b**) Normalized elastic moduli of metal carbon materials from references indicated in the data legend. Stiffening due to e-beam curing is indicated by an orange dashed arrow. The additional gallium metal content for FIB deposits was not considered.

Figure 17 replots data from Figure 16 for which the metal content of FEBID and FIBID materials could be inferred together with the Hashin–Shtrikman expressions for the elastic moduli of composites [107]. We refer the reader to their work as their expressions are too lengthy to be reproduced here. The elastic moduli of the Pt-C materials cover a large range. Using the Hashin–Shtrikman expressions for elastic moduli in composite materials, it can be shown that small amounts of stiff or compliant carbon matrices considerably strengthen or weaken the deposit’s elastic modulus with respect to the metal elastic modulus. When the matrix is very compliant as for amorphous carbon and polymeric material, even high metal contents above 50 at.% may not considerably stiffen the nanocomposite according to the lower Hashin–Shtrikman bound, as can be seen in Figure 17a with the blue curve. Inversely, 30 at.% of a stiff diamond-like carbon matrix considerably strengthens a compliant metal structure by a factor of around 2, taking the upper Hashin–Shtrikman bound for nanocomposites; see the orange curve. This is very interesting from the view of being able to independently tune the functionality given by the metal content (electrical, magnetic, plasmonic) and the mechanical functionality. From Figure 15, metal percolation thresholds needed for close-to-metal-bulk functionality (conductivity, magnetism, etc.) are around 60 to 70 at.%. With the potential complimentary 30–40 at.% carbon matrix, the composite modulus could thus be increased (diamond-like matrix) or lowered (polymer-like matrix) over two orders of magnitude within the Hashin–Shtrikman upper/lower bounds indicated.

Figure 17b summarizes all metal–carbon data. A few state-of-the-art features can be spotted, (i) as-grown low metal content FEBID material exclusively populates the (*E/E_metal_* < 0.2; *x* < 20%) space, (ii) EBC of FEBID low metal content material as well as FEBID conditions with a high e-dose will stiffen the carbonaceous matrix, even to such an extent that the metal modulus is exceeded, see Figure 17a, (iii) the low metal content FIBID material populates the (*E/E_metal_* > 0.2; *x* < 10%) space, meaning that the as-deposited FIB carbonaceous matrix is stiffer than the typical as-deposited FEB matrix. EBC or high e-dose FEBID conditions are needed for FEBID to get into the same parameter space. (iv) Interestingly, with around 50 at.% metal content, materials start to have a stiffer carbonaceous matrix, which lifts them up into the (*E/E_metal_* < 0.8; 40% < *x* < 50%) space. The high-metal content FEBID and FIBID elastic modulus space still awaits more systematic investigation and thus research in electron/ion-triggered dissociation of organometallic molecules into pure metal.

### 3.2. Hardness 

**Figure 18:**
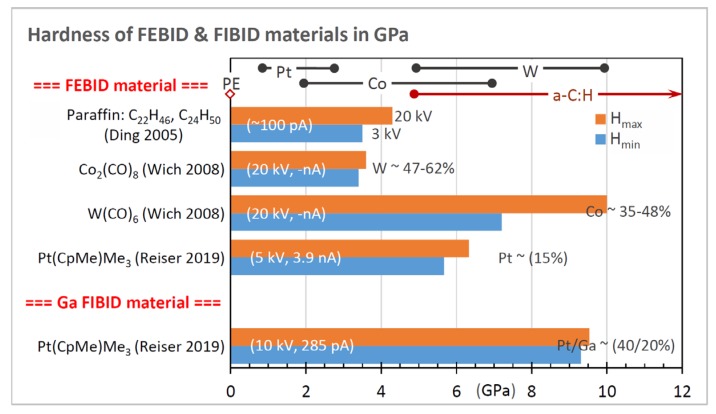
Reported nanoindentation hardness values for FEBID and FIBID material. The characterization method, beam acceleration voltage, and beam current are reported together with the metal content. Metal contents are given in at.%. Hardness ranges for the metals and amorphous hydrogenated carbon are also indicated.

As was outlined in Section 2.2.2, hardness is a measure of material resistance to localized plastic deformation, which can be induced by nanoindentation. Nanoindentation requires relatively large few micron-sized sample volumes, and the scarce data obtainable so far is summarized in Figure 18. 

Nanoindentation hardness ranges were indicated in Figure 18 for Pt [108], W [109], Co [110], Ga_2_O_3_ [111], amorphous hydrogenated carbon [112], and polyethylene [113]. The nanoindentation hardness ranges of amorphous hydrogenated carbon were measured to vary from about 5 GPa (40 at.% H) to 21 GPa (30 at.% H) [112], while the polyethylene populates the low range from 0.022 GPa to 0.073 GPa, depending on density and molecular weight [113]. Nanoindentation hardness values of tetragonal amorphous carbon range from 20 GPa (65% sp^3^) to 54 GPa (90% sp^3^) [114], and the highest values are about 88 GPa [115]; however, these were not shown in Figure 18, as they were out of range. The hardness measured for FEBID carbon deposits from paraffin by Ding et al. [45] is close to the low range of amorphous hydrogenated carbon and may contain some more polymeric contributions, which make it softer. The ratios of hardness to elastic modulus H/E are 0.07 and 0.1 for 3 keV and 20 keV electron energy, respectively. Interestingly, H/E = 0.1 is the ratio found for diamond, while diamond-like films have ratios closer to 0.16 [92]. Turning to the metal–carbon materials, the FEBID cobalt–carbon and tungsten–carbon materials with metal contents from roughly 40–60 at.% have nanoindentations hardnesses within the pure metal range. Then, the contribution of the carbonaceous matrix to hardness is probably in the same range, and it may be a bit softer as the minimum measured nanocomposite hardnesses tend to the lower metal hardness range. An interesting effect is seen for the FEBID 15 at.% Pt-carbon material; its hardness is above the platinum hardness range, meaning that here, the hardness of the FEBID carbonaceous material was dominant. For the Ga-FIBID material, the carbonaceous matrix has a smaller amount of 40 at.%, which must compensate the soft platinum and gallium fraction. The nanocomposite hardness falls into the gallium oxide range; however, this may be accidental. The ratios of hardness to elastic modulus H/E ranges for the platinum–carbon material are 0.08–0.11 for FEBID and 0.07–0.1 for FIBID, which was also found for the carbon FEBID deposit of Ding et al. [45] and hints at a relatively hard carbon matrix dominating the hardness in nanocomposites obtained by FEBID and FIBID with soft metal containing organometallic precursors.

### 3.3. Quality Factors 

**Figure 19:**
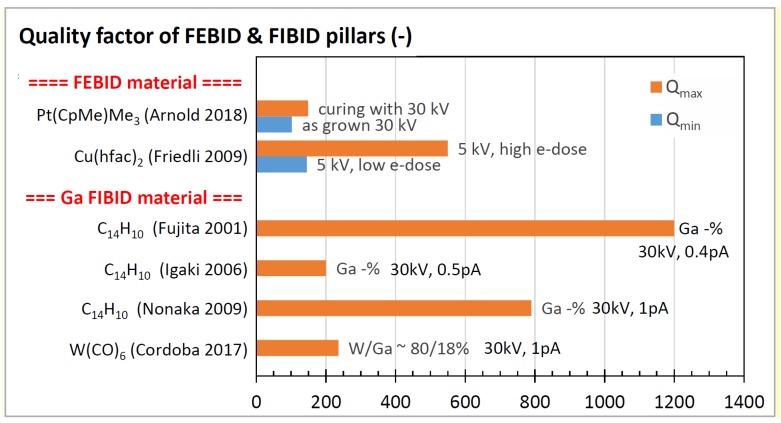
Reported quality factors for FEBID and FIBID pillars from vacuum, room temperature vibration experiments in the range of 0.1 to 1 MHz.

Quality factors appear as output of resonance measurement, as shown in Figure 8b. The vibration amplitude and the phase shift as functions of the excitation frequency of a piezoelectric actuator contain the information about the quality factor. The full width at half maximum (FWHM) of the resonance peak is a measure for the quality factor $Q=\sqrt{2}f_{1}/FWHM$. The broader the resonance peak, the smaller the quality factor, signifying that damping (energy loss) is involved in the vibration. Friedli et al. [53] pointed out that in general, the acquired SE signal due to the pillar deflection is not linear with the pillar’s vibration amplitude due to edge effects generating artefacts in the SE signal and due to the limitation of the dynamic range at low amplitudes resulting from the SE signal’s strong dependence on the FEB position relative to the deposit. Hence, errors are inherently introduced when measuring the frequency peak full width at half maximum and determining the quality factor with the previous formula. However, the related quality factor Q of the vibrating pillar can be precisely calculated from the slope dϕ/df of the phase vs. frequency diagram at resonance via $Q=0.5f_{0}\cdot d\phi /df$. A quality factor of *Q* = 274 at *f_0_* = 566.6 kHz at room temperature resulted from the phase vs. frequency diagram, as shown in the bottom of Figure 8b. As quality factor measurements of FEBID or FIBID pillars are mostly performed in vacuum environments, external energy loss due to molecular, viscous, or turbulent ambient gas flow can be neglected, and thus the damping can be attributed to internal energy losses, such as internal friction (viscosity) or clamping loss (connection to substrate). As a comparison, quality factors at 300 K were measured to be around 6680 (*f_0_* = 14.2 MHz) for polycrystalline diamond, 10^5^ (*f_0_* = 4.9 MHz) for monocrystalline silicon, and 3.8∙× 10^5^ (*f_0_* = 13.2 MHz) for monocrystalline diamond resonators [116] in vacuum conditions. As the quality factor is frequency-dependent, values that are above it will increase [117] for the 1 MHz range where typical FEBID and FIBID resonance experiments were performed. The lower quality factors summarized in Figure 19 for FEBID and FIBID pillars can be attributed to the non-monolithic nature of the interface between the Si substrate and pillar, the amorphous versus mono/polycrystalline nature, as well as the nanogranular metal–carbon matrix inner structure of the FEBID/FIBID pillars. It can be anticipated that the smaller the quality factors in FEBID and FIBID materials, the larger the polymer/hydrogen content in the carbon network that leads to damping of the vibration. Furthermore, the inherent core–shell structure of FEBID and FIBID pillars, see Figure 2, may also hide the high-quality factor diamond-like core material due to the soft/compliant outer shell.

### 3.4. Yield and Fracture Strength 

**Figure 20:**
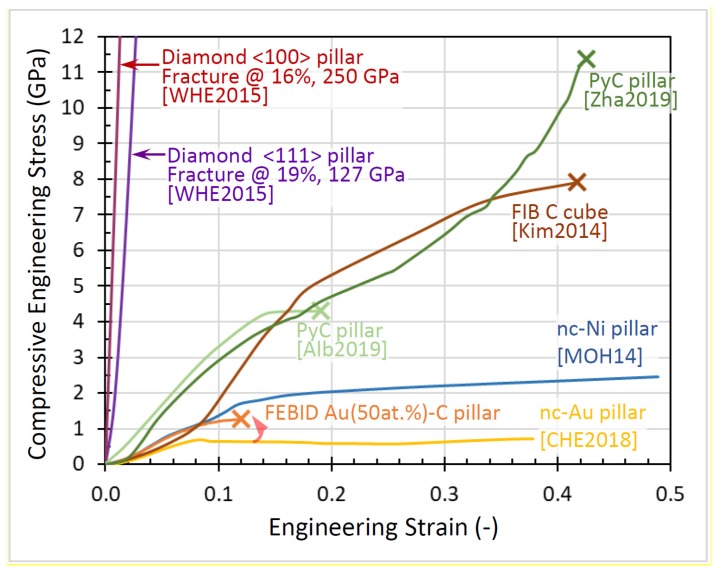
Compilation of pillar nanocompression experiments of varying materials: single crystal diamond [120], pyrolytic graphite (PyC) [118,119], nanocrystalline nickel (nc-NI) [122], nanocrystalline gold (nc-Au) [121], FEBID Au-C pillar (this work), and an FIB carbon cube of amorphous hydrogenated carbon [41]. The crosses signify the appearance of cracks or catastrophic fracture. Note the ductility of all pillars in contrast to the stiff and brittle diamond pillars.

As discussed in Section 2.2.3. plastic deformation including fracture strength (stress at fracture) can be captured in nanocompression experiments with blunt flat indenter tips monitoring force and displacement. This has been performed for metal, diamond, and pyrolytic carbon (PyC) pillars recently, while data on FEBID and FIBID pillars are scarce for now. Pyrolytic carbon has a relatively low density between around 1 and 2 g/cm^3^ with an sp^2^ bond fraction of around 90%, thus being a representative of amorphous (hydrogenated) carbon a-C(:H) material with respect to the ternary phase diagram in Figure 10. Pillars with varying density can be obtained by two-photon polymer lithography in photoresist and a pyrolysis step (heating to about 800–900 °C in vacuum for some hours). The volume shrinks considerably during the pyrolysis step and is in the order of 90%. According to the PyC preparation method, fracture strength and strain can vary widely, as shown in Figure 20 for two examples from Zhang et al. [118] and Albiez et al. [119]. Generally, the pillar diameter had an influence on ductility and fracture performance; smaller diameter pillars contained less volume defects, which made them tougher, showing higher fracture strength at high strains. The PyC pillars from Zhang et al. [118] are the most ductile *and* strong material reported so far, which withstand 40% compressive strain without catastrophic failure (although cracks start to form). Wheeler et al. [120] showed that diamond pillars have elastic behavior until catastrophic fracture; however, their fracture strength of 127 GPa and 250 GPa for <100> and <111> orientation, respectively, is not shown on the scale of Figure 20. However, diamond is brittle and fractures at strains < 20%. Nanocrystalline metal pillars populate the opposite region with large strains and low fracture strength due to their ductility, which comes with the ability to accommodate deformations plastically by several mechanisms, such as dislocation formation and gliding, grain boundary sliding and rotation, as well as twinning. Figure 20 shows examples of electrodeposited gold pillars from Chen et al. [121] and of nickel pillars from Mohanty et al. [122]. For metal pillars, not only the size of the pillar is important, but also the metal grain size. Strengthening as a function of the metal grain size is known from the Hall–Petch relation, while also an inverse Hall–Petch relation was observed, which is responsible for the softening of nanograin metal material. For a recent overview on these mechanisms, the reader is referred to [123]. Consequently, when comparing pillar nanocompression experiments, the diameter and the internal pillar microstructure needs to be characterized, when drawing conclusions about the origins of material improvement. The presently available two FEBID and FIBID pillar examples of this work and of Kim et al. [41] in Figure 20 fall into the ductile metal and graphitic carbon/a-C(:H) range, respectively. Obviously, there is still a large parameter space toward diamond awaiting to be explored for FEBID and FIBID metal–carbon material in the context of fracture strength and toughness.

**Figure 21:**
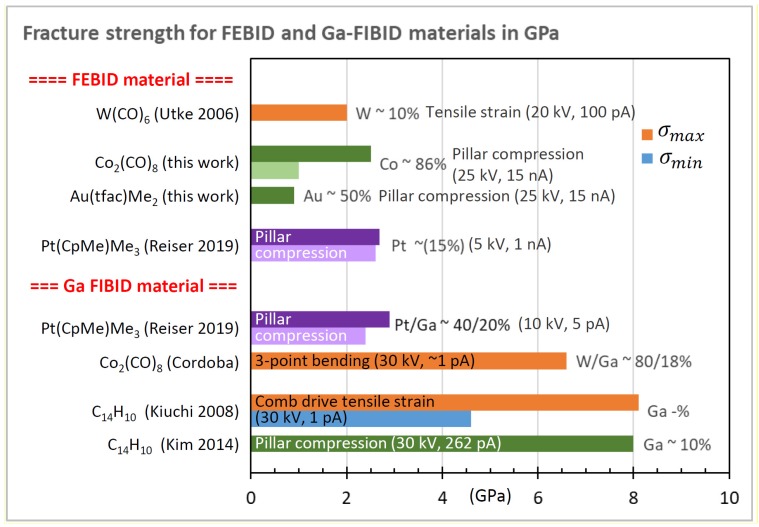
Fracture strengths of as grown FEBID and FIBID structures. The methods are indicated as well as the metal content whenever available from the reference. The precursor molecules and the first authors are listed on the vertical axis. Green colors highlight measurements on large volume structures; other colors indicate measurements on high aspect ratio pillars with potential core–shell internal structure. The purple bars indicate the measured stress at 7% strain and not the fracture stress, which is higher.

The fracture strengths of FEBID and FIBID materials are summarized in Figure 21. 

According to Tabor [124], the yield stress Y, hardness H, and elastic modulus E for solids can be related via Marsh’s equation

(17) H/Y≅0.07+0.6 ln(E/Y)

However, the present data situation does not allow to check whether FEBID/FIBID materials obey this relation. Another observation was noted by Lewis et al. [48]. Repeating nanoindentation loading–unloading cycles, they observed a hysteresis pointing to the viscoelasticity of the FEBID platinum–carbon material deposited under their specific conditions; see Figure 13c. They assigned a polymeric nature to the FEBID carbonaceous matrix to explain their findings. 

### 3.5. Density

**Figure 22:**
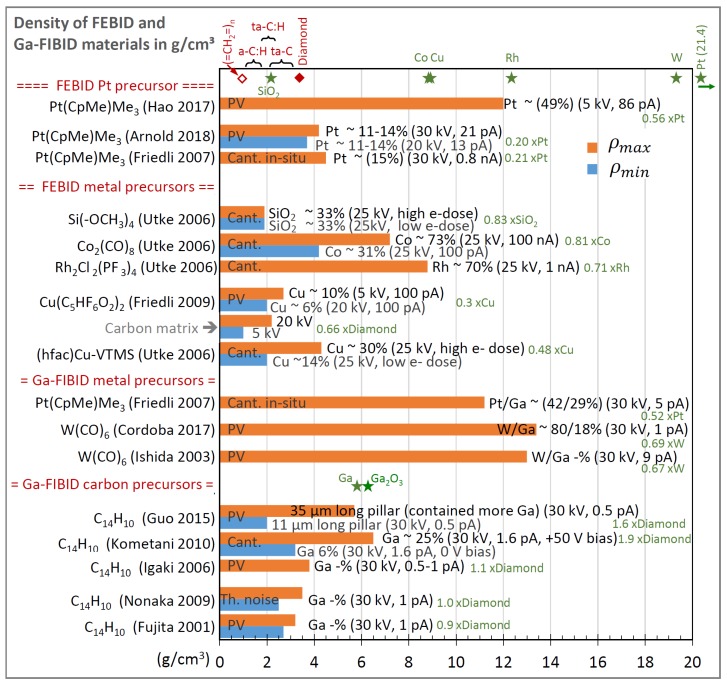
Summary of density measurements of FEBID and FIBID (noted as FEB and FIB) grown materials. The vertical axis lists the precursor molecule used to grow the structure and the author reference; if not noted otherwise, the density is reported for as-grown material. Maximum and minimum densities as far as accessible, the metal contents, and the measurement methods are indicated. Metal contents given in parentheses were interpolated in the respective reference but not explicitly measured. The measurement methods are pillar vibration (PV), cantilever-based (Cant.), and thermal noise (Th. Noise). Bulk density values of pure compounds and elements are also indicated. The density ratios of deposit to bulk are noted in green for the maximum deposit density.

Measurement methods for the density determination of nanostructures confine to the pillar vibration method (which requires the determination of the elastic modulus according to Equation (14) and the mass determination via cantilever-based frequency shift methods in the picogram range; see Figure 8 in Section 2.2.3. All available data are reported in Figure 22 together with bulk metal density values for comparison. The carbonaceous matrix density can reach the value of 3.5 g/cm^3^ at its highest, which corresponds to the density of diamond. Friedli et al. [53] and Utke et al. [55] backcalculated the density of the carbonaceous matrix from metal–carbon FEBID material and concluded that matrix densification is best achieved at high electron energy and a high electron dose.

The density of a compound A_x_B_1-x_ can be estimated by a linear relation between the densities of the pure constituents $\rho_{A}$ and $\rho_{B}$

(18) $$\rho_{A_{x}B_{1-x}}=x\rho_{A}+\left(1-x\right)\rho_{B}$$

**Figure 23:**
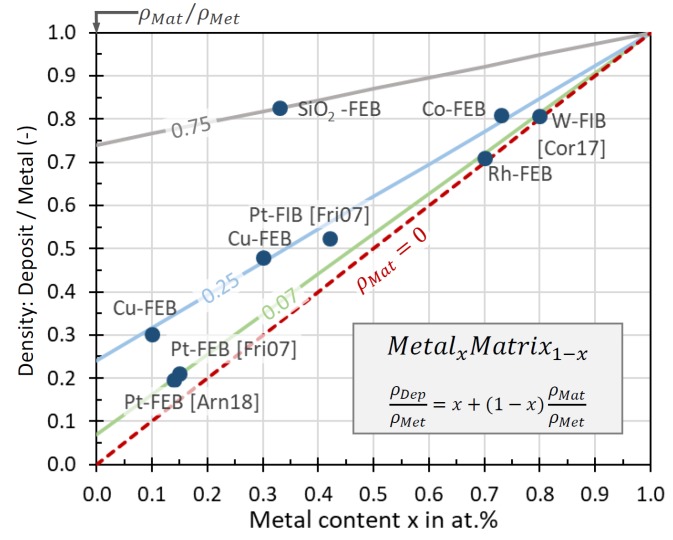
Normalized density of FEBID and FIBID material vs. reported metal content. The lines were obtained from Equation (18) normalizing to the metal density (see inset). Numerical values refer to the corresponding $\rho_{Mat}/\rho_{Met}$ ratios; the metal is shown in the data point legends.

With known metal densities $\rho_{Met}$, the matrix densities $\rho_{Mat}$ can be calculated. For the FEBID platinum–carbon, copper–carbon, and SiO_2_–carbon materials, the matrix densities of 1.5, 2.2, and 1.4 g/cm^3^ were obtained, respectively. Two copper FEBID materials obtained from Cu(hfac)_2_ and (hfac)Cu-VTMS situate close to the same line ($\rho_{Mat}/\rho_{Met}=$0.25, i.e. $\rho_{Mat}=2.2\;g/cm^{3}$) and have the same comparable matrix density. This may hint to the (hfac) ligand fragments as the main component of the matrix, as both precursors have them in their molecule. However, more data would be needed to draw firm conclusions about this. The same can be seen for the Pt FEB deposits from Arnold et al. [47] and Friedli et al. [46], which have the same matrix density of 1.5 g/cm^3^ while the Pt-FIB deposit arrives at a very high carbonaceous matrix density of about 4 g/cm^3^ (not shown in Figure 23), which is due to the gallium implantation. 

## 4. 3D Structures in Mechanical Experiments

**Figure 24:**
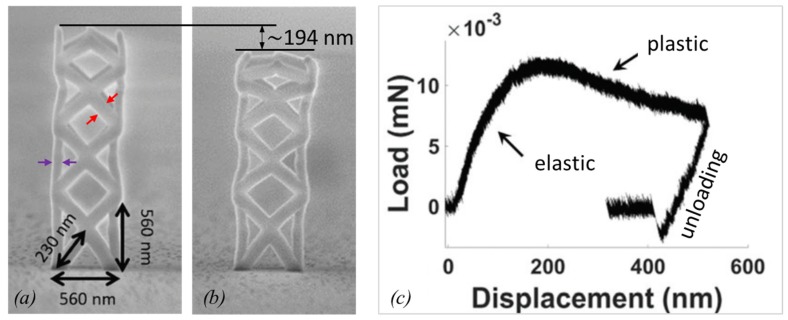
Compression along the vertical axis of a 3D FEBID tower truss lattice structure. (**a**) Initial lattice dimensions. Note the different dimension of the zigzag units in front view (red arrows) and projected view (purple arrows). (**b**) Dimensions after 500 nm compression along the long lattice axis. Note the plastic deformation of 194 nm. (**c**) Monitored force–displacement curve during a tower truss compression experiment with 400 nm plastic deformation. Modified from Lewis et al. [48].

In this section, we review the results obtained on three-dimensional FEBID structures. An example of a nanocompression experiment of a tower lattice structure [48] is shown in Figure 24. On all four sides, the lattice is composed of the same zigzag structure seen from the front view. Comparing the projected view of the left and right sides (the zigzag structures are seen just as straight pillars) with the front view dimensions shows that the cross-section of the composing zigzag units is elliptical. This is a consequence of scanning the focused electron beam to obtain a freestanding pillar with a given slope. The elliptic cross-section can be varied by adjusting the vertical deposition rate in relation to the horizontal scan speed [125]. The lattice was compressed by 500 nm using a large flat nanoindenter punch and then released. At around 180 nm indentation, there is plastic deformation of the lattice, which can be witnessed comparing Figure 24a,b with each other. Correspondingly, the force displacement curve in Figure 4c shows an initial (close to) linear slope, which is expected for the elastic region and a yield part where the load is decreasing in the slope and then in value due to plastic deformation.

The linear slope is a measure for the elastic modulus of the entire tower architecture; to obtain an elastic modulus, the force would need to be converted into mechanical stress by assigning an effective area to it. For individual straight pillars, this area is $\pi r^{2}$; however, for connected multipillar composed architectures, the cross-sectional area of the top face of the structure is taken. 

**Figure 25:**
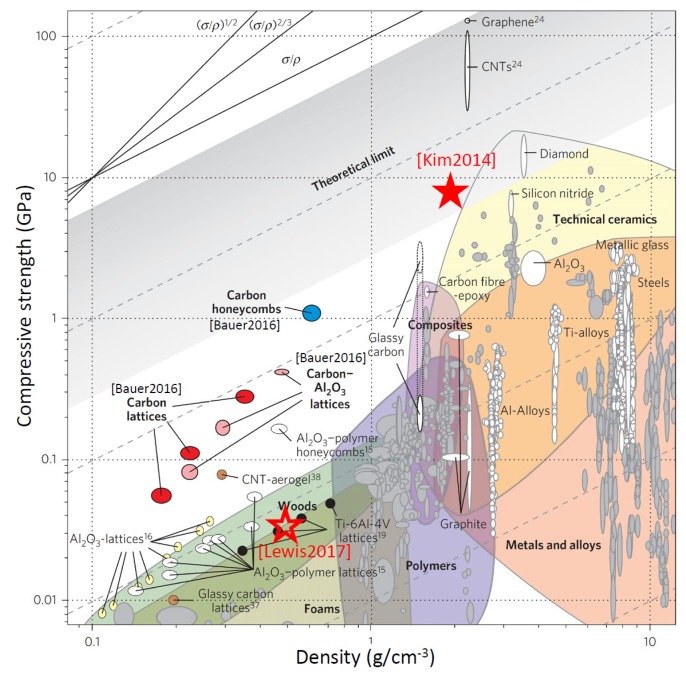
Strength–density scatter plot. The open asterisk denotes the FEBID Pt–C lattice tower structure from Lewis et al. [48] shown in Figure 24 and the full asterisk denotes the Ga–FIBID carbon material from Kim et al. [41] shown in Figure 4f. Modified from Bauer et al. [127].

**Figure 26:**
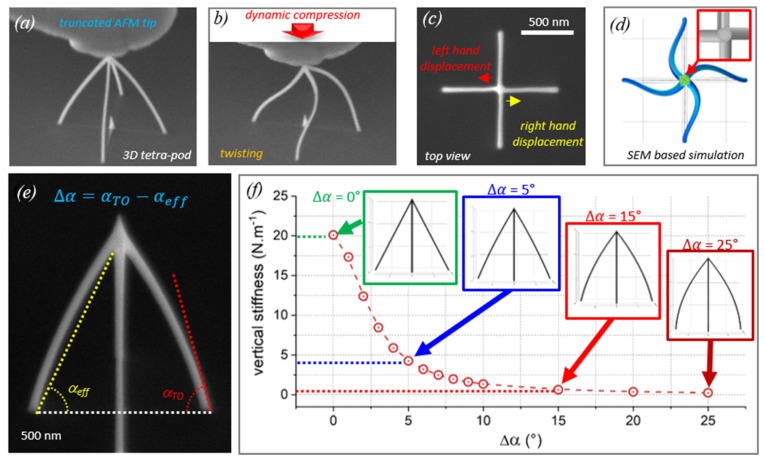
Nanofabrication-related implications on mechanical properties. (**a**) shows an SEM side-view of a Pt-C tetrapod fabricated via 3D FEBID on SiO_2_. The upper part is a truncated AFM tip, which allows dynamic compression of such nanoarchitectures. The latter is shown in (**b**), where unexpected twisting effects were stochastically found. The SEM top view image in (**c**) reveals slight mismatches in the merging zone by left/right-handed displacements in the sub-10 nm regime (see arrows). (**d**) shows a finite-element simulation during vertical compression, including the aforementioned displacement as shown in the red-framed inset. This mismatch, although small, induce the twisting effect, which clearly shows the high demands on spatial precision during 3D nanofabrication. (**e**) shows another tetrapod structure in an SEM side view, in which the non-straight branches become evident. For further studies, Δα was defined as indicated in blue. Then, this parameter was varied in finite-element simulations, while vertical stiffnesses were calculated as shown in (**f**) and further validated by experiments (see ref [128]). As evident, even a small Δα of 5° implies a four-fold decrease in stiffness, which decays quickly with larger Δα. As for the upper row, these experiments reveal the high demands on nanofabrication to fully exploit the intended mechanical properties. Images were reproduced with permission from reference [128].

Scatter plots, or so-called Ashby charts in mechanics [126], are generally used to compare the mechanical properties of material families. Figure 25 shows a compressive strength-density scatter plot from Bauer et al. [127] complemented with FEBID and FIBID material data obtained from the literature. Densities < 1 g/cm^3^ are typically populated by porous material, such as wood or foams, and by designed porous lattice structures, as for example shown in Figure 24. The aim of the designed porous lattice architectures is to keep a maximum of strength at a minimum of weight (density). The theoretically achievable limit is given by a linear scaling law of strength on density (straight lines with unit slope in Figure 25) with upper and lower bounds for carbon defined by graphene and diamond. Several glassy carbon-based lattice architectures fabricated by two-photon polymer lithography and controlled pyrolysis were investigated by Bauer et al. [127]. Their glassy carbon bulk material had an elastic modulus of about 22 GPa, and the honeycomb structures figure close to the theoretical lower bound limit of diamond. The only mechanically measured Pt-C FEBID lattice from Figure 24 is roughly comparable in average density to Bauer et al.’s honeycomb structures; however, its strength is about 50 times lower. This is due to the lower elastic modulus of the bulk FEBID Pt-C material (around 8 GPa), some cross-section irregularities of the lattice itself, and its zigzag units, which shear under compression rather than taking the load axially. However, all of these weaknesses are technical and could be addressed nowadays by FEBID; see also Figure 26. The Ga–FIBID bulk carbon material of Kim et al. [41] is within the theoretical lower bound scaling limit specified by diamond and could potentially shift toward it with higher density applying proper e-beam curing conditions, for example.

A more recent example of nanomechanical measurements was demonstrated by Sattelkow et al. [128], who used 3D FEBID for the fabrication of freestanding 3D nanoarchitectures in the frame of AFM-based thermal nanoprobes. The authors used a combined approach between finite element simulations and SEM assisted, in situ AFM compression experiments, which revealed the strong implications of the overall design and the fabrication accuracy. In more detail, four-legged tetrapods with heights around 1.5 μm and total opening angles of 30° revealed vertical stiffnesses > 60 N/m, which is about an order of magnitude higher than the targeted AFM cantilever stiffnesses. The radial stiffness, which is as important during AFM operation, was found in the range of 5 N/m with a high radial symmetry for 4 or more legs. However, the striking findings of that study were the occasional observations of (i) twisting effects and (ii) non-linear force–distance behavior during dynamic compression. Correlated studies could unravel the former effect as a consequence of spatial fabrication mismatches in the topmost merging zone. Although small (< 5 nm), the implications were massive, as representatively shown in Figure 26a–d. The non-linear force–distance behavior could be traced back to non-straight single branches, which induce off-axis flexing with strong implications on the vertical stiffness, as shown in Figure 26e,f. Both effects could be successfully minimized by adapting the process conditions (straight branches) and different design in the merging zone (larger merging volumes) and successfully applied as stable AFM nanoprobes. The study clearly reveals that the small dimensions, achievable via 3D FEBID, increase the demands on fabrication accuracy to fully exploit their nanomechanical properties. For that, this technology is ideal, as it allows fast design adaptions toward ideal conditions. 

## 5. Irradiation Parameter Influence on Mechanical Properties

In this section, we now focus on the implications of continuous growth on already deposited material. The situation is similar to e-beam curing or the irradiation of polymers in e-beam lithography. Here, the governing parameters are the primary electron/ion energy, the transferred energy, the dissipated energy, and the irradiation dose.

The maximum energy $E_{max}\;$that an electron with primary energy $E_{0}$ can transfer to an atom via knock-on collision is in the non-relativistic case [10]

(19) $$E_{max}≅4\frac{m_{e}}{m_{a}}E_{0}$$

where $m_{e}$ and $m_{a}$ are the electron and atom mass, respectively. We obtain 65 eV for hydrogen, 5.4 eV for carbon, 4.1 eV for oxygen, and 3.4 eV for fluorine for maximum transferred energies by 30 kV electrons. They compare to bond dissociation energies for C-H with ≈ 4–4.5 eV, C-C with ≈ 3.6–3.9 eV, C≡O with ≈ 11.2 eV, and C-F ≈ 5 eV [129]. Such bonds would need to be broken to release hydrogen, oxygen, and fluorine from hydrogenated, oxygenated, and fluorinated carbonaceous matrices. We remind the reader that such matrix compositions result from co-deposited organic ligands used to render the metal volatile for gas injection. Examples of carbonaceous matrix compositions from methylcyclopentadienyl, carbonyl, and hexafluoro-acetylacetonate ligand fragments are summarized in Table 1. At 30 keV electron irradiation, it appears that hydrogen bonds can be broken as well as carbon can be rearranged. Any oxygen or fluorine, if present in the carbonaceous matrix, are not likely to be removed. Furthermore, atom displacement still requires additional energy for hopping in the carbon network or bond re-arrangement for carbon network reticulation (sp^3^ formation). Thus, a higher primary electron energy will result in stiff carbon networks. For example, transferred energies at 300 kV acceleration voltage, as typical for TEM, will be around 40 eV (fluorine) to 650 eV (hydrogen) and cause a densification/stiffening at a much higher rate compared to 30 kV acceleration voltage, which is the highest primary electron energy for most SEMs. Here, volatile atoms can be either removed or lead to the formation of amorphous sp^3^ carbon networks from former hydrogen (oxygen, fluorine) sp^2^ bonds. 

We speculate that prolonged TEM observation in the experiments of Hao et al. [90] was the probable cause of the extraordinary stiff carbon matrix, as shown in Figure 16. In line with the proposed stiffening for higher electron beam energies, Ding et al. [45] found increasing elastic moduli and hardness for FEBID bulk material grown from phenanthrene. Friedli et al. [53] found increasing densities and elastic moduli in pillars with increasing primary electron energies for the same electron dose. For ion beams, the electron mass in Equation (19) needs to be replaced by the mass of the impinging ions (gallium, helium, etc.). As the masses of colliding ions and affected atoms are far more similar than for electrons, the maximum transferred energies during ion bombardment at 30 kV are in the low keV range. That provides sufficient energy for breaking any hydrogen, oxygen, or fluorine bonds but as well for amorphization of the carbon network and sputtering. 

The implanted energy (or power times time) is responsible for heating up the specimen and dissipating thermal energy for carbon network formation reactions as a kind of local pyrolysis in the irradiated area as well as mobility to volatile species in the matrix to escape. In other words, moderate higher temperatures will favor a clean carbon matrix (with low hydrogen, oxygen, or fluorine content) and a proper network arrangement, leading to stiff carbon materials. For bulk samples, the temperature increase was roughly proportional to the product of acceleration voltage and beam current, while for pillars, the confined dimensions resulted in a more complex expression, as discussed in Section 2.1. 

The electron dose is defined as the beam current times time per irradiated area, and it is widely used in the field of e-beam and ion beam lithography, when exposing a polymer resist to a two-dimensional irradiation area pattern. The same concept can be widened to FEBID and FIBID thin film deposits and to 3D structures, which are irradiated during electron beam curing (after growth). For a given beam energy and beam current, Arnold et al. [47] found a slightly non-linear elastic modulus increase with irradiation time during the electron beam curing of their pillars, which was attributed to a structural change in the carbonaceous matrix, as discussed in Section 3.1.2. They measured a five-fold increase during 50 min of curing, although saturation in the Young modulus was not found after that time.

To estimate the average electron dose of the exposed deposit during the FEBID process, Utke et al. [55] introduced the parameter dose per deposited atom in units of impinging electrons per deposited atom, which is the inverse of the deposition yield. For higher doses, they found a densification of the deposits by a factor around 2. To explain these findings, one needs to understand that e-beam curing happens to a certain extent already during FEBID/FIBID growth. Depending on growth speed and electron trajectories, the deposit volume below the growth front is subjected to a certain accumulating number of electrons. The higher the dose per deposited atom, the more e-beam curing happens already during growth. As a high dose per deposited atom relates to low deposition yield (or speed), e-beam curing can be considered as an additional process for in situ tuning, although the settings have to be adapted carefully to exploit the full potential of this approach. 

## 6. Conclusions

This article reviewed the portfolio of nanomechanical measurement methods used to determine elastic modulus, hardness, quality factors, yield and fracture strength, and density of nanoscale and microscale 3D structures directly written by gas-assisted focused electron and ion beam-induced deposition. Due to their small dimensions, SEM (or TEM) integrated setups with actuators or force sensors are employed in arrangements of compression, tension, bending, buckling, and vibration with the nanoscale or microscale objects. Deviations from idealized geometries were pointed out as well as the necessity to employ finite element methods for proper analysis.

Vertical high aspect ratio nanopillars, grown via stationary beams during FEBID or FIBID, mostly lead to stiff core–compliant shell structures. The qualitative and quantitative core–shell characteristics strongly depend on primary energies, subsequently generated secondary electrons, and the trajectory behavior (interaction volume), which strongly differs for ions and electrons. The softer shell mostly ranges between 10 and 70 nm and can be removed by post-growth plasma treatments to access the stiff core. When scanning the focused electron or ion beam over a larger footprint to form micron-sized "bulk" structures, the internal structure homogenizes in terms of composition and mechanics, while the contribution of the peripheral surfaces on the overall mechanical property measurement can be minimized. Whenever substantiated by proper literature, the article also discussed the implications of ion implantation, arising temperatures, and overall doses to generate a more general picture of the situation.

FEBID and FIBID materials obtained from organic carbon precursors as well as the carbonaceous matrix obtained from the co-deposition of the organic ligands from metalorganic precursors can be well described within the ternary sp^3^–sp^2^–hydrogen phase diagram, which was derived from studies of the hard carbon coating community. The potential of FEBID and FIBID to tune the elastic moduli of pillars from the compliant polymer range over amorphous (hydrogenated) carbon a-C(:H) and tetragonal amorphous hydrogenated carbon ta-C:H toward diamond-like ta-C material from volatile organic carbon compounds/volatile metalorganic precursors was clearly demonstrated. However, quantitative measurements of carbon sp^3^/sp^2^ hybridization fractions and absolute hydrogen (and implanted gallium) contents are very scarce. Clearly, it requires dedicated studies to link hybridization and the composition of carbon networks to electron and ion irradiation to growth/curing/post-growth treatment parameters for the controlled tuning of mechanical properties. 

Although FEBID and FIBID have demonstrated their potential to grow pure or at least high metal content materials for selected elements, the bulk of mechanical measurements so far were performed for so-called nanogranular (or nanocomposite) materials with metal contents of 10–20 at.%, which are embedded as nanocrystals in a hydrogenated, oxygenated, or fluorinated carbonaceous matrix. No data were available to answer the question regarding which of the carbonaceous matrices (hydrogenated, oxygenated, fluorinated, or a mix of the former) has better mechanical performance, and, to this respect, which precursor will lead to the best mechanical performance of FEBID and FIBID material. The Hashin–Shtrikman framework for the elastic moduli of nanocomposites showed that the mechanical properties of the carbonaceous matrix dominate the nanocomposite material and predicts that even for metal contents higher than 80 at.%, stiffening or softening by the carbonaceous can be expected. Supporting experimental studies would be highly relevant, as they might open a route to tune the core functionalities (electrical, magnetic, optical, superconducting) relatively independently from the elastic modulus. Densities of FEBID and FIBID materials can be treated with the rule of mixture between the extremes of the metal and carbonaceous matrix. Of note is that the density of carbonaceous material can only be directly measured for FEBID, because FIBID always entails ion implantation.

Data become even scarcer for FEBID and FIBID material concerning quality factors, hardness, and strength. Especially after fabrication, the quality factors (inversely related to vibration damping) are comparably low and require more focused studies to clarify the reasons. The hardness typically ranges from amorphous hydrogenated carbon a-C:H as the lower end toward values larger than the pure (polycrystalline) metal. This is promising as it again contains the potential to open up a route for independent mechanical optimization apart from metal functionality and vice versa. Fundamental studies on the influence of size effects on materials strength is currently a hot topic for the broad mechanics community. Here, the FEBID and FIBID community can contribute to the synthesis of 3D lattices with low volume density and tuned metal/matrix content determining the mechanical and functional properties of the composing lattice units and the overall lattice as such. First strength measurements indicate that materials with pyrolytic carbon or metal nanopillars can be synthesized. However, it can be anticipated from this review that the potential of FEBID and FIBID can go further toward diamond-like material and the orthogonal tuning of functionalities, which was not yet demonstrated by other nanoprinting methods, due to the very different working principles of focused electron/ion beam-induced deposition. Hence, it can be concluded that FEBID/FIBID-based 3D nanoprinting contains huge, partly unexplored potential for material tuning in a diverse range of applications, which exploits the full potential in combination with its true direct-write character, executable on practically any given material and surface morphology. 

## Figures and Tables

**Table 1:** Summary of precursors and references of focused electron beam and focused ion beam-induced deposition (FEBID and FIBID) materials for which mechanical properties were reported. Chemical formulae and abbreviations as used in this article are given as well as the resulting deposit metal content and matrix composition. The maximum achieved metal contents (sometimes with additional co-reactant gases) obtained so far are shown in comparison with the corresponding reference. The abbreviation a-C:H,O,F reads as amorphous carbon being hydrogenated, oxygenated, and/or fluorinated.

Element with Precursor, Linear Formula, Sum Formula	Abbreviation	Metal (at.%) *	Matrix *	Max Metal Content Reported so far
C-FIBID with phenanthrene [36,37,38,39,40,41,42,43,44], C_14_H_10_	C_14_H_10_	3–25 Ga	a-C:H_x_	Up to 25 at% Ga contamination depending on deposition parameters
C-FEBID with paraffin (C_n_H_2n+2_) [45] : n-docesane C_22_H_46_; n-tetracosane, C_24_H_50_	C_22_H_46_, C_24_H_50_	0	a-C:H_x_	Cleanest—no metal in deposit when using FEBID
Pt-FEBID and FIBID with Trimethyl- (methylcyclopentadienyl)–platinum [46,47,48,49], (CH_3_)_3_Pt(C_5_H_4_CH_3_); PtC_9_H_13_	Pt- (CpMe)Me_3_	10–15 (FEB) Pt/Ga: 42/20 (FIB) [46]	a-C:H_x_	FEB: Pt ≈100 at.% (+H_2_O) [26] FIB: ≈ 45 at% (5 at.% Ga) [50]
W-FEBID and FIBID with Tungsten-hexacarbonyl [51,52], W(CO)_6_	W(CO)_6_	W/Ga: ≈ 80/18 (FIB) [51] W: 47–62 (FEB) [52]	FIB: a-C:O, ≈ C_85_O_15_ [51] FEB: a-C:O, ≈ C_80_O_20_ [52]	FIB: ≈ 80 at.% W [51] FEB: ≈ 62 at.% [52]
Co-FEBID with Dicobaltoctacarbonyl Co_2_(CO)_8_ (This work and [52])	Co_2_(CO)_8_	≈ 72 (This work) ≈ 42 [52]	a-C:O, ≈ C_67_O_33_ (This work)a-C:O, ≈ C_80_O_20_ [52]	Co ≈ 95 at.% (FEB) [21]
Au-FEBID with Dimethyl(trifluoro- acetylacetonate)-gold (This work), (CH_3_)_2_Au(O_2_C_5_H_4_F_3_); C_7_H_10_AuF_3_O_2_	Au(tfac)Me_2_	50 (FEB)	a-C:O,F,H ≈ C_85_O_20_H_x_	FEB: Au 100 at.% (+H_2_O) [32]
Cu-FEB: Copper(II) hexafluoroacetyl- acetonate [53], Cu(C_5_HF_6_O_2_)_2_; CuC_10_H_2_F_12_O_4_	Cu(hfac)_2_	6–10 (FEB)	a-C:O,F,H ≈ C_60...70_O_20..22_F_4..8_H_x_	FEB: ≈ 10 at.% [54] No FIB data
Cu-FEBID with hexafluoropentane- dionate-Copper–vinyltrimethylsilane [55], C_5_H_12_Si-Cu-C_5_HF_6_O_2_; CuC_10_H_13_F_6_O_2_Si	(hfac)Cu- VTMS	14–30 (FEB)	a-C:O,F,H,Si≈ C_70_O_14_Si_10_H_x_ [56]	FEB: ≈ 95 at.% [57]FIB: ≈ 95 at.% Cu (5 at.% Ga) [58] Both achieved with heating
Fe-FIBID with Ferrocene [59], Fe(C_5_H_5_)_2_; FeC_10_H_10_	Fe(Cp)_2_	5 at% Fe (FIB)	a-C:H_x_	FEB: 95 at.% with Fe(CO)_5_ [60,61]
SiO_2_-FIBID with Tetramethylcyclotetra- siloxane [62], (HSiCH_3_O)_4_; Si_4_C_4_H_12_O_4_	(HSiCH_3_O)_4_	5 at% Ga [63]	C < AES noise level [63]	FIB: 95 at.% SiO_2_, 5at.% Ga (+traces of O_2_) [63]
SiO_2_-FEBID with Tetramethoxysilane (This work), Si(OCH_3_)_4_; SiO_4_C_4_H_12_	Si(OCH_3_)_4_	33 (FEB)	a-C:H,O ≈ C_56_O_44_H_x_ [34]	FEB 100 at.% SiO_2_ (+H_2_O) [34]
Rh-FEBID with Rhodium-tetrakis- chlorotrifluorophosphine (This work), [RhCl(PF_3_)_2_]_2_; Rh_2_Cl_2_P_4_F_12_	Rh_2_Cl_2_(PF_3_)_4_	70 (FEB)	a-P:Cl ≈ P_77_Cl_23_F_0_ [64]	FEB: 60–70 at.% (This work) No FIB data

* Literature references in these two columns differ from column one (left) when no composition data was found there.
